# All around suboptimal health — a joint position paper of the Suboptimal Health Study Consortium and European Association for Predictive, Preventive and Personalised Medicine

**DOI:** 10.1007/s13167-021-00253-2

**Published:** 2021-09-13

**Authors:** Wei Wang, Yuxiang Yan, Zheng Guo, Haifeng Hou, Monique Garcia, Xuerui Tan, Enoch Odame Anto, Gehendra Mahara, Yulu Zheng, Bo Li, Timothy Kang, Zhaohua Zhong, Youxin Wang, Xiuhua Guo, Olga Golubnitschaja, On Behalf of Suboptimal Health Study Consortium and European Association for Predictive, Preventive and Personalised Medicine

**Affiliations:** 1 Centre for Precision Health, Edith Cowan University, Perth, Australia; 2 Beijing Key Laboratory of Clinical Epidemiology, Capital Medical University, Beijing, China; 3 School of Public Health, Shandong First Medical University & Shandong Academy of Medical Sciences, Tai’an, China; 4 First Affiliated Hospital, Shantou University Medical College, Shantou, China; 5 Suboptimal Health Study Consortium, Kumasi, Ghana; 6 Suboptimal Health Study Consortium, Perth, Australia; 7 Suboptimal Health Study Consortium, Beijing, China; 8 Suboptimal Health Study Consortium, Bonn, Germany; 9 European Association for Predictive, Preventive and Personalised, Medicine, Brussels, Belgium; 10 Department of Medical Diagnostics, College of Health Science, Kwame Nkrumah University of Science and Technology, Kumasi, Ghana; 11 School of Nursing and Health, Henan University, Kaifeng, China; 12 Institute of Chinese Acuology, Perth, Australia; 13 School of Basic Medicine, Harbin Medical University, Harbin, China; 14 Predictive, Preventive and Personalised (3P) Medicine, Department of Radiation Oncology, University Hospital Bonn, Rheinische Friedrich-Wilhelms-Universität Bonn, Bonn, Germany

**Keywords:** Predictive preventive personalised medicine (PPPM/3PM), Suboptimal health status (SHS), Traditional medicine, Body mass index (BMI), Multi-level diagnostics, Omics, Glycan, Liquid biopsy, Modifiable preventable risks, Risk assessment, Communicable, Non-communicable diseases, Adolescence, Stress overload, Cardiovascular disease, Cancers, Neurologic diseases, Mood disorders, Microbiome, Periodontal health, Natural substances, Lifestyle, Dietary habits, Behavioural patterns, Sleep medicine, Individualised patient profile, Artificial intelligence (AI), Multi-parametric analysis, Big data management, Medical ethics, Health economy, Health policy, Epidemics, Pandemics, COVID-19

## Abstract

First two decades of the twenty-first century are characterised by epidemics of non-communicable diseases such as many hundreds of millions of patients diagnosed with cardiovascular diseases and the type 2 diabetes mellitus, breast, lung, liver and prostate malignancies, neurological, sleep, mood and eye disorders, amongst others. Consequent socio-economic burden is tremendous. Unprecedented decrease in age of maladaptive individuals has been reported. The absolute majority of expanding non-communicable disorders carry a chronic character, over a couple of years progressing from reversible suboptimal health conditions to irreversible severe pathologies and cascading collateral complications. The time-frame between onset of SHS and clinical manifestation of associated disorders is the operational area for an application of reliable risk assessment tools and predictive diagnostics followed by the cost-effective targeted prevention and treatments tailored to the person.

This article demonstrates advanced strategies in bio/medical sciences and healthcare focused on suboptimal health conditions in the frame-work of Predictive, Preventive and Personalised Medicine (3PM/PPPM). Potential benefits in healthcare systems and for society at large include but are not restricted to an improved life-quality of major populations and socio-economical groups, advanced professionalism of healthcare-givers and sustainable healthcare economy. Amongst others, following medical areas are proposed to strongly benefit from PPPM strategies applied to the identification and treatment of suboptimal health conditions:

- Stress overload associated pathologies
- Male and female health
- Planned pregnancies
- Periodontal health
- Eye disorders
- Inflammatory disorders, wound healing and pain management with associated complications
- Metabolic disorders and suboptimal body weight
- Cardiovascular pathologies
- Cancers
- Stroke, particularly of unknown aetiology and in young individuals
- Sleep medicine
- Sports medicine
- Improved individual outcomes under pandemic conditions such as COVID-19.

## Introduction

### Epidemics of non-communicable diseases in the twenty-first century

First two decades of the twenty-first century are characterised by epidemics of non-communicable diseases such as many hundreds of millions of patients diagnosed with cardiovascular diseases (CVD) [1] and the type 2 diabetes mellitus (T2DM) [2]; some cancer types such as breast, lung, liver and prostate malignancies [3, 4]; and neurological, sleep, mood and eye disorders [5], amongst others. Consequent socio-economic burden is tremendous.

### Unprecedented decrease in age of disordering individuals

Currently observed, there is an unprecedented in the age of individuals disease on non-communicable pathologies [5]. To this end, teenagers present a great portion of new cases diagnosed with T2DM annually [2]. There is an increasing prevalence of preventable eye disorders in high school students [6]. Mood disorders and suicide are frequently monitored in youth [5] as well as young strokes (15–50 years of age) with unknown aetiology [7]. Aggressive metastasising cancers are increasing reported for 20 + years old patients with particularly poor outcomes [4, 8–10].

### Paradigm shift from reactive to predictive preventive and personalised medicine (3PM)

The paradigm shift from reactive medical services to predictive approach, targeted prevention and personalisation of treatments has been proposed by the European Association for Predictive, Preventive and Personalised Medicine (EPMA, Brussels, www.epmanet.eu) to reverse above documented trends, to improve overall quality of medical services making them available for any socio-economic group in the society [11–15].

### Modifiable risks and preventable illnesses: focus on suboptimal health measures

**Fig. 1:**
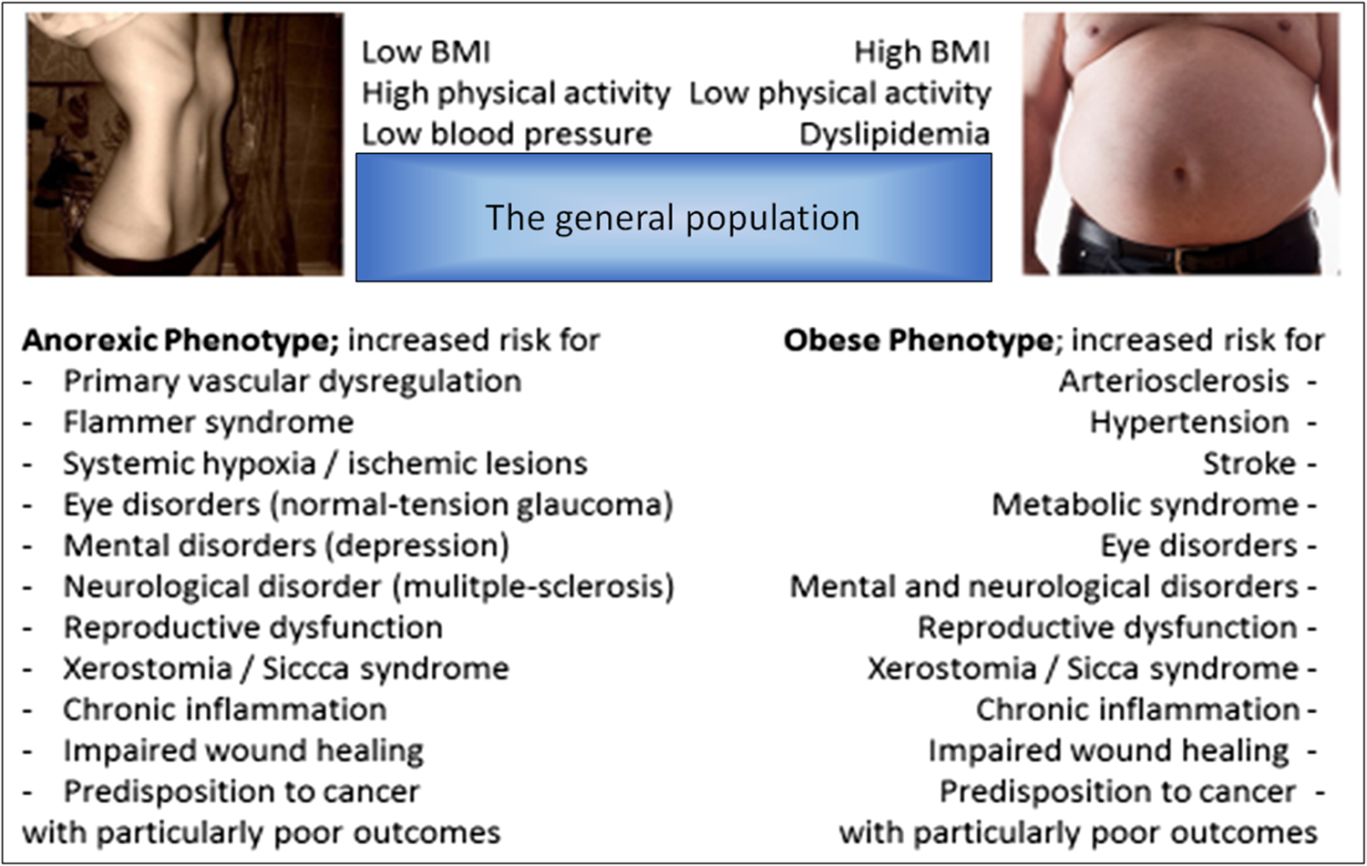
Anorexic versus obese phenotype: the paradox of the health risks similarity; the figure is adapted from [5]

Particularly modifiable risk factors are instrumental for targeted cost-effective prevention of illnesses in the population. To this end, inadequate lifestyle and dietary habits are most prominent examples of modifiable risks strongly associated with numerous of developing and progressing pathologies. Several parameters are indicative for that such as individually suboptimal (both overweight and underweight) body weight. Figure 1 highlights pathologies frequently associated on one hand with anorexic and on the other hand with obese phenotypes.

Currently applied weight loss programmes raise questions and concerns regarding individually adapted recommendations and individual health benefits for persons undergoing such as programme. To this end, a recently study performed in the USA which involved 20,002 adults has analysed the efficacy of receiving weight-related advice from healthcare professionals: surprisingly, patients who reported receiving weight-related advice, achieved worse weight outcomes a year later, compared to unsupervised patients [16]. Per evidence, individually optimal weight as one of the critical health parameters demonstrates the basic principle of personalised medicine, namely “ONE SIZE does not FIT ALL” being strongly dependent on the individual genetic predisposition, geographic origin, cultural and nutritional habits, amongst other lifestyle measures.

Health, as defined by the World Health Organisation, is “a state of complete physical, mental, and social well-being and not merely the absence of disease or infirmity” (WHO) [17]. For many, that level of health remains elusive -there is an increasing number of individuals worldwide who report general malaise even in the absence of a diagnosable disorder [18]. A full evaluation of health conditions is necessary for the effective implementation of public health intervention [19].

However, there are persons who complain poor healthy perception but not in diagnosable disorders. This intermediate state between health and a diagnosable disease is known as suboptimal health status (SHS) [20, 21].

SHS is a reversible borderline condition between optimal health and diseases, characterised by declines in vitality, physiological function and capacity for self-adaptation, which does not meet any criteria of diseases but suffer from the perception of health complaints, general weakness and low energy. Individuals with SHS have predispositions to physical or mental diseases, especially non-communicable diseases. The majority of non-communicable disorders have a chronic feature by progressing over a couple of years from a reversible suboptimal health condition to irreversible pathology with corresponding complications [22].

On behalf of the Suboptimal Health Study Consortium/SHSC, this multiple centre dedicated working group provides a deep analysis on the topic SHS from the perspectives of Predictive, Preventive and Personalised Medicine (PPPM) considering the multifaceted aspects of both “health care” and “disease prevention” practices including (1) definition of SHS; (2) identification and quantification of reversible damage to the health; (3) the status quo of suboptimal health predictive diagnostic tools; (4) avoiding of under- and over-diagnosis with significant economic impacts to healthcare; (5) suboptimal health status, risk factors and risk group; (6) individualised preventive measures; (7) treatment algorithms tailored to the person; and (8) outlook: conventional and traditional medicine — a “hand-in-hand” collaboration benefiting the patient and healthcare at large.

## Definition of SHS

Suboptimal* Health Status* (SHS) is “an overall physical status between health and illness characterised by the perception of health complaints, chronic fatigue, and a constellation of physical symptoms such as the cardiovascular system, the digestive system, the immune system, and mental status; lasting for at least 3 months” [18, 21]. Although SHS is not a disease state, several studies have suggested that SHS might precede the occurrence of chronic diseases, including cardiovascular diseases (CVD) [23, 24] and type 2 diabetes mellitus (T2DM) [25, 26]. From the perspective of predictive, preventive, and personalised medicine (PPPM), the concept of SHS reflects the viewpoint that chronic diseases can be effectively predicted and prevented before the occurrence of a clinical manifestation of severe pathologies [27]. PPPM is an integrative concept in the health care sector that enables to predict individual predisposition before the onset of disease, to provide targeted preventive measures and to create personalised treatment algorithms tailored to the person [5, 15]. Chronic diseases, such as CVD and T2DM, are treated after disease onset, which is a very much delayed approach in terms of PPPM. As a subclinical, reversible stage of chronic diseases, SHS plays a significant role in the prediction and prevention of chronic diseases in terms of PPPM [5].

SHS may not necessarily represent the condition known as either early stage or preclinical period of illness, but it is commonly defined as the period preceding the occurrence of clinical manifestations of diseases [19–21]. Individuals in early stage or preclinical period usually need the interventions with specific therapies that enable to prevent or delay the onset of the disease. People with SHS are considered to require no specific clinical treatment but may be at a high risk for some diseases. It has been reported that only 23% people in SHS sought for medical help, and nearly 80% of individuals who complained of SHS-related symptoms showed no improvements within 6 months [28]. It is, therefore, urgent to promote the actions to identify individuals with SHS from the perspectives of PPPM [29].

SHS, by reference to its characteristics, is understood to be associated with the following syndromes: general weakness, unexplained medical syndrome (UMS), chronic fatigue syndrome (CFS), myalgic encephalomyelitis (ME), post-viral fatigue syndrome (PVFS), Flammer Syndrome, and chronic fatigue immune dysfunction syndrome (CFIDS) [18, 30]. To improve the healthcare services from the perspective of PPPM, more studies are needed to clarify the SHS-associated health damages and dysfunctions, by which individuals exposed to SHS-induced damage will receive specific remedy.

**Fig. 2:**
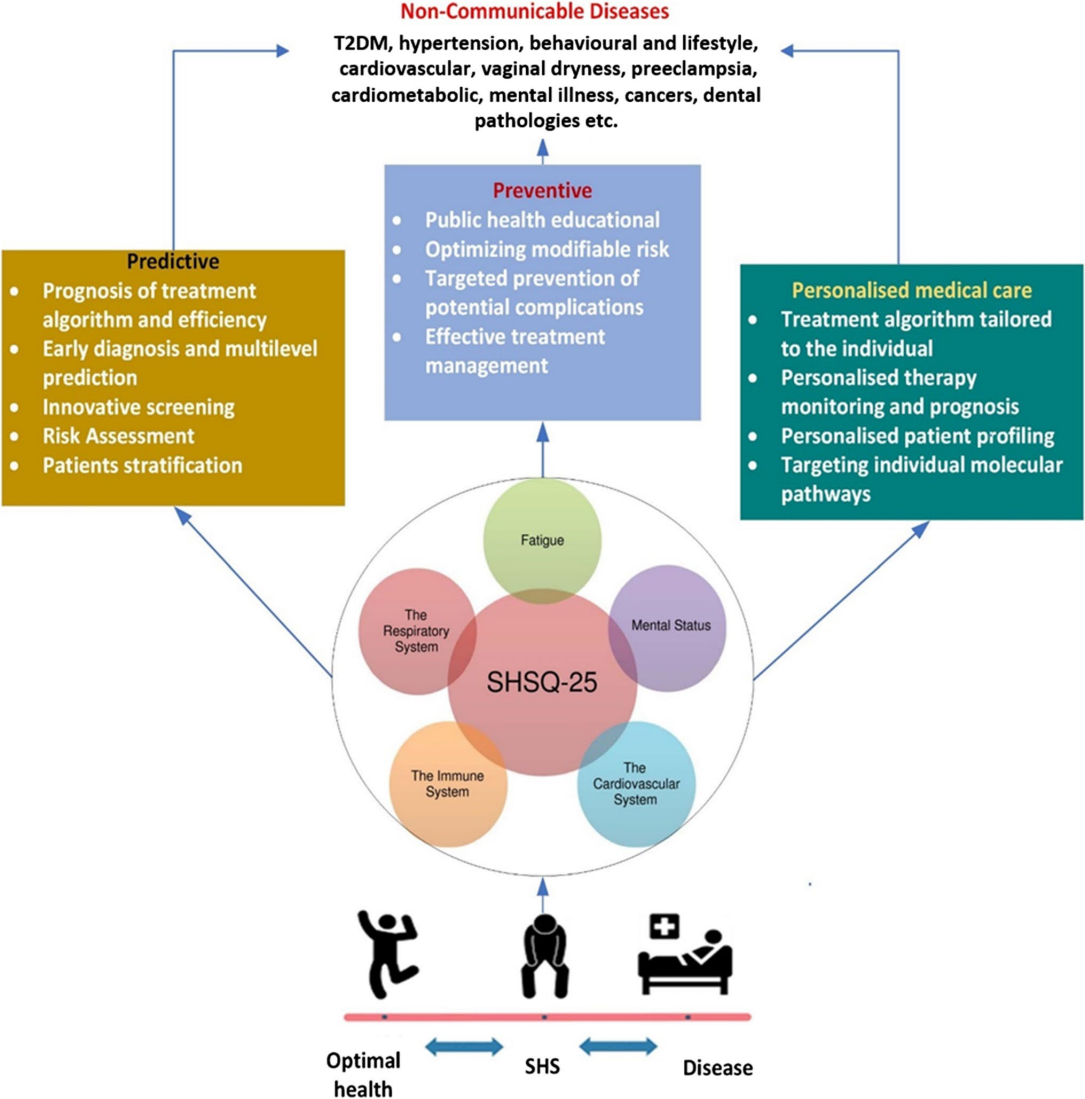
Suboptimal health status is instrumental for predictive preventive and personalised medical care tailored to the person vulnerable to non-communicable diseases

A non-invasive screening tool for measuring SHS has been developed: suboptimal health status questionnaire-25 (SHSQ-25), and it has been validated in African [31, 32], European [24], and Asian populations [27, 29, 33]. The SHSQ-25 accounts for the multidimensionality of SHS by encompassing five domains: fatigue, the cardiovascular system, the digestive system, the immune system, and mental status, with 25 supporting elements [29]. Figure 2 highlights SHS as being crucial to advance predictive preventive, and personalised medical care tailored to the person vulnerable to non-communicable diseases.

Since early detection followed by appropriate intervention is important for prevention from the onset of diseases, it should be requested for clinicians to shift from the perspective of delayed intervention approaches to early screening of individuals with SHS [31]. The heterogeneity and uncontrollability of health-related perceptions and inconsistent phenotypes of health complaints make it difficult to discriminate the persons in SHS from general populations. Commonly, identification of the five domains of SHS is of interest for health improvement: fatigue, cardiovascular system, digestive tract, immune system, and mental state [20, 27].

## Established approaches to identify and quantify reversible damage to health

Health scales and questionnaires are effective measurement instruments for recognition of reversible damage to the health that are difficult to be measured directly [27]. During the past decades, health scales and questionnaires have been extensively used in measurement of various aspects of health such as different symptoms or the presence of a particular trait [34, 35], such as the “Suboptimal Health Status Questionnaire-25 (SHSQ-25)” [27], “Cornell Medical Index (CMI) [36]”, and “12-item General Health Questionnaire (GHQ-12)” [37] et al. The SHSQ-25 was developed for investigating suboptimal health status (SHS), a reversible damage condition to the health [27]. The SHSQ-25 includes 25 items and encompasses 5 subscales: fatigue, the cardiovascular system, the digestive tract, the immune system, and mental status. Based on the influence of chronic stress on health, the preliminary SHSQ-25 was developed by methods including literature review, focus group discussions and feedback from relevant medical staff such as physicians, health managers, pathophysiologists, and clinical epidemiologist. The questionnaire was tested in a large cross-sectional investigation confirmed the validity and reliability of the questionnaire [27].

### The procedure of questionnaire development

The main procedure of questionnaire development includes: concepting → item → questionnaire. Before designing the questionnaire, the purpose of the research and theoretical hypothesis should be determined. The items of a newly developed questionnaire can come from several sources, such as established instruments, literature, focus group discussions, and expert opinions [27, 34]. After the items have been written, focus groups and expert panels are used again to discuss whether these items are relevant, clear, unambiguous, written in terms that are understood by potential respondents. After that, a preliminary health questionnaire is constructed based on the item pool. The investigator should consider the sequence of questions, logical structure, convenience for answer, and possible psychological impact on the respondents. Usually, a pilot study is performed to assess whether the questions are relevant and feasible for use. Based on the feedback of the respondents in the pilot study, the questionnaire is then revised, and the final version is formed.

To obtain valid responses, an appropriate scoring system for quantifying the responses should be used. It is suggested that using as many classes as they can be used for responses of health questionnaire [38]. Rating scales, which seem to have been first formalised by Likert, are widely used in the health and social sciences in the assessment of variables that cannot be measured directly [39, 40]. For example, all questions of the SHSQ-25 asked the individual to rate a specific statement on a five-point Likert-type scale (never or almost never, now and then, often, very often, and always) how often they suffered from various specific discomfort in the preceding 3 months [27].

### Assessment of reliability of health scale

The two most fundamental and important characteristics of any measurement procedure are reliability and validity. A good measurement instrument should be stable and reliable, and the results of measurement should be reproducible. Reliability refers to the extent to which a scale or questionnaire produces the same results using the same instruments under similar conditions [41]. There are two aspects of reliability, namely: external reliability and internal reliability. External reliability usually refers to the consistency of measurement tools at different time. Internal reliability refers to if a measurement instrument measures a single concept, that is, the extent of internal consistency amongst the items of the instrument. There are different indices to measure reliability including test–retest reliability, alternate form reliability, split-half reliability, and Cronbach’s alpha et al. Cronbach’s alpha is a widely used measure reliability that assesses the degree to which the items are related to each other; it measures a unified construct [42]. A Cronbach’s alpha coefficient greater than 0.70 are generally considered acceptable for group comparisons, and 0.90 for person-level comparisons [43].

### Assessment validity of health scale

Validity is defined as the extent to which the instrument measures what it purports to measure [9]. There are two aspects of validity, namely: internal validity and external validity. Internal validity generally refers to the accuracy of inferences about whether one variable cause another. External validity refers to what extent can our results are able to accurately generalised to other participants or situations. There are several types of validity, including content validity, criterion validity, construct validity, discriminant validity and convergent validity. Construct validity is widely used to assess the validity of a given questionnaire, which reflect the degree to which an instrument measures the trait or theoretical construct that it is intended to measure. To establish the construct validity, investigator has to generate predictions based upon the hypothetical construct, and these predictions can be then tested to give support to the validity of the questionnaire [44]. Statistically, construct validity is usually tested through exploratory or confirmatory factor analysis [45, 46].

In summary, a well-developed health instrument is essential for predictive, preventive, and personalised medicine. To be useful, health measurement scales should be reliable and valid, and convenient for respondents to use.

## Persons at high versus low risk: avoiding under- and over-diagnosis with significant economic impacts to healthcare

Assessment of SHS has demonstrated the ability to predict the risk of certain chronic diseases, such as cardiovascular disease (CVD) [24]. Identification of persons at risk of developing chronic disease is an outcome shared by both SHS and Predictive, Preventive and Personalised Medicine (PPPM) [5, 27]. PPPM is a concept enabling the prediction of a person’s predisposition to disease before its onset, followed by preventive measures delivered via personalised treatment algorithms [13]. Reducing the prevalence of chronic diseases is urgent, especially considering that the estimated total global cost of CVD will increase from US$863 billion (2010) to over US$1044 billion by 2030 [47].

The under- and over-diagnosis of chronic diseases, while dichotomous, both augment the substantial economic burdens to healthcare by virtue of their respective sequelae [48]. Under-diagnosis is “the failure to recognise or correctly diagnose a disease or condition especially in a significant proportion of patients” [49], whilst over-diagnosis occurs when an individual is diagnosed with a disease that would have otherwise never caused them symptoms, harm, or early death, but are then exposed to treatments where the risks outweigh the benefits [48, 50]. In this paragraph, we demonstrate how assessment of SHS identifies persons at high versus low risk: avoiding under- and over-diagnosis with significant economic impacts to healthcare, evidencing SHS as the new gold standard in the global fight against chronic disease from the perspectives of PPPM.

### Under-diagnosis

Effective screening of chronic diseases, such as T2DM or prediabetes, is key in PPPM quest to curb the prevalence of non-communicable diseases [13]. Early identification enables prompt treatment and delays long-term complications [25]. This is especially important considering T2DM has entered the top 10 causes of death worldwide [51], with a projected trajectory for its prevalence to reach 500 million by 2025–2030 [15]. Furthermore, T2DM is prognosticated to reach pandemic proportions in 10–20 years, contributing to a potential “economic disaster of health care systems on a global scale” [5]. Despite this, T2DM is still frequently under-diagnosed [52]. A valid and cost-effective screening tool is paramount [27]. Amongst the new methods of screening instruments, SHS deserves special attention [27].

*Case study one* — Incorporation of suboptimal health status as a potential risk assessment for type 2 diabetes mellitus: a case control study in a Ghanaian population

A cross-sectional study conducted in Ghana incorporated SHS as a potential risk assessment for T2DM [25]. Patients with diagnosed T2DM and healthy controls (*n* = 241:264) respectively, completed the SHSQ-25 questionnaire, and were concurrently assessed for anthropometric, clinical, and biochemical parameters [25]. In healthy individuals, SHS was able to discriminate between highly at-risk individuals and low risk individuals (median score > 21 represents high SHS (poor health) whereas median score < 21 represents low SHS (good health)) [25]. This study revealed both systolic and diastolic blood pressure to be significantly associated with high SHS scores [25], an alarming finding considering high blood pressure is the main risk factor or T2DM and CVD [25]. Moreover, undiagnosed hypertension was common amongst the participants [25]. In an ideal setting, and in line with PPPM, receiving a high SHS score should prompt individuals to have their clinical/biochemical markers tested at a healthcare centre. Further, an individual with a high SHS score may have undiagnosed, asymptomatic T2DM, or its related co-morbidities, that requires urgent intervention or treatment. In either case, identification of person’s at high risk through SHS provides the opportunity for preventive treatment by way of dietary/lifestyle modifications prescribed by healthcare professionals. The study also found that amongst T2DM sufferers, there was poor management of risk factors, with the majority of T2DM patients having higher than recommended levels of fasting plasma glucose and HbA1c levels (i.e. > 7 mmol/l and > 7.2 mmol/l, respectively) [25]. Many of these patients are at risk of developing co-morbidities and complications as a result of inadequate management [25].

*Case study two* — Suboptimal health status as an independent risk factor for T2DM in a community-based cohort: the China suboptimal health cohort study

The China Suboptimal Health Cohort Study (COACS) is a longitudinal study initiated in 2013 to understand the impact of SHS on the progress of T2DM [21]. A recent prospective study from the COACS performed a baseline evaluation of 3635 participants in 2013–2014, consisting of a medical history, standardised physical evaluation (such as measuring BMI and blood pressure) laboratory assessment of T2DM (such as fasting glucose level and total cholesterol), and measurement of SHS using the SHSQ-25 [53]. T2DM outcomes were then followed up annually from 2013 to 2017 [26]. The study aimed to compare the difference in cumulative incidence between SHS groups and also investigated the relationship between SHS levels and risk of T2DM onset [26]. Over the course of the study, 61 participants developed T2DM, whilst 3574 remained in good health. Using logistic regression models, SHS scores were entered as quartiles (Q), with the lowest quartile as the reference [26]. The results showed that participants with higher levels of SHS had a significantly higher risk of T2DM [16]. Participants in the highest quartile of SHS had a 1.7-fold risk of developing T2DM when compared to the lowest quartile [26]. Compared with the lowest level of SHS (Q1), the Q4, Q3, and Q2 were found to be associated with 1.7-, 1.6-, and 1.5-fold risks of developing T2DM, respectively [26], indicating that the risk will increase with the increasing SHS performance of an individual [26]. The potential applicability of SHS as dynamic monitoring index for the development of T2DM should be considered [26]. Table 1 highlights relative risk of T2DM by quartile group of SHS.

**Table 1:** Relative risk (RR) of T2DM incidents by quartile groups of SHS (26): SHS avoids under-diagnosis by identifying persons at high versus low risk, with significant economic impacts to healthcare; model 1, crude model; model 2, adjusting for age and gender; model 3, model 2 + age, gender, smoking status, body mass index (BMI), systolic blood pressure (SBP), diastolic blood pressure (DBP), total cholesterol (TC), triglycerides (TG), low-density lipoprotein cholesterol (LDLC), and high-density lipoprotein cholesterol (HDLC); model in female, model 2 + age, BMI, SBP, DBP, TC, LDLC, and HDLC. Statistically significant RRs are presented in italics

Models	RRs of SHS (95% CI)			
	Q1 (SHS < 8)	Q2 (SHS = 8–14)	Q3 (SHS = 14–24)	Q4 (SHS > 24)
Model 1	1.00 (ref)	*1.80 (1.20–2.72)*	*1.98 (1.33–2.96)*	*1.93 (1.29–2.88)*
Model 2	1.00 (ref)	*1.61 (1.08–2.48)*	*1.71 (1.13–2.56)*	*1.80 (1.17–2.66)*
Model 3	1.00 (ref)	1.54 (0.95–2.29)	*1.62 (1.02–2.41)*	*1.71 (1.11–2.61)*
Stratified analysis				
Model in male	1.00 (ref)	*1.72 (1.02–2.92)*	*1.72 (1.02–2.91)*	*1.73 (1.01–2.98)*
Model in female	1.00 (ref)	1.69 (0.86–3.34)	1.90 (0.99–3.64)	*1.93 (1.02–3.65)*

The aforementioned examples demonstrate how SHS avoids the under-diagnosis of chronic diseases, in this case T2DM, by identifying and stratifying persons at risk [25, 26]. In developing countries such as Ghana, routine screening for SHS by adoption of SHSQ-25 would serve to overcome the economic barriers associated with a lack of laboratory tests and treatments, facilitating PPPM, thus having a significant impact to healthcare.

The under-diagnosis of T2DM is not limited to developing countries such as Ghana and China, however, with developed nations also severely affected. For example, the cost of undiagnosed diabetes in the USA in 2017 was estimated to be $31.7 billion dollars, with prediabetes costing $43.4 billion [53]. The high cost of treating undiagnosed diabetes underscores the importance of screening for early detection and management [54] yet despite the screening recommendations by the American Diabetes Association (individuals over 45 and at-risk younger adults every three years) [55] between 2005 and 2012, less than half of these individuals were actually screened; a result attributed to the social determinants of health [56]. Compared to many other survey instruments, the SHSQ-25 is a short, simple, and inexpensive tool to use; the implementation of which should be adopted as part of routine screening programs in both developing and developed nations. This will serve to avoid the high levels of under-diagnosis for chronic diseases, such as T2DM, through the identification of persons at high versus low risk with significant economic impact to healthcare.

### Over*-diagnosis*

Over-diagnosis has multiple negative effects on both patients and cost burden for healthcare systems; patients are exposed to unnecessary tests and treatments, and resources are wasted that could be better spent treating or prevent genuine illness [48]. The cost of over-diagnosis is staggering, draining healthcare systems of billions of dollars each year [48]. For example, an estimated $200 billion is squandered on unneeded treatments in North America each year [48]. Over-diagnosis rests on the premise that diagnosis confers no benefit [57]. For example, diagnosis of a small, low-grade prostate cancer in an elderly man as a result of screening that leads to unnecessary treatments is considered over-diagnosis, as the cancer would never have caused problems for the patient in his lifetime [57].

Suboptimal health status rests on the premise that diagnosis does offer a benefit. Detecting SHS enables patient stratification by predicting an individual’s predisposition to disease, giving clinicians the opportunity to implement targeted preventive measures before the actual onset of disease [18]. Identification of person’s at high versus low risk using SHS therefore avoids over-diagnosis, demonstrated in the examples below.

*Case study three* — Association of suboptimal health status and cardiovascular risk factors in urban Chinese workers

A recent study analysed the association of SHS and cardiovascular risk factors in urban Chinese workers and found participants with high SHS scores had a higher risk of CVD than those with low scores [29]. Compared to the low SHS group, systolic and diastolic blood pressure, plasma glucose, total cholesterol, triglyceride levels, and BMI were significantly higher amongst the high SHS group (*P* < 0.001). In addition, serum cortisol levels were much higher amongst the high SHS group than amongst the low SHS group (204.31 versus 161.33 ng/ml, *P* < 0.001) [29]. SHS is associated with cardiovascular risk factors and contributes to the development of cardiovascular disease [29]. Table 2 highlights relevance of SHS for cardiovascular disease.

**Table 2:** Comparison of the cardiovascular risk factors between high and low SHS score group (29)

Cardiovascular risk factors	SHS score “high”, mean ± SD	SHS score “low”, mean ± SD	*T test*	*P value*^*a*^
SBP (mmHg)	119.43 ± 13.27	115.31 ± 13.19	8.573	< 0.001
DBP (mmHg)	77.57 ± 7.38	75.38 ± 7.89	7.880	< 0.001
GLU (mmol/L)	5.23 ± 0.57	5.17 ± 0.55	2.941	< 0.001
TCH (mmol/L)	4.48 ± 0.76	4.32 ± 0.78	5.708	< 0.001
TG (mmol/L)	1.17 ± 0.58	1.08 ± 0.46	4.709	< 0.001
HDLC (mmol/L)	1.32 ± 0.32	1.36 ± 0.36	-3.230	< 0.001
LDLC (mmol/L)	2.82 ± 0.70	2.78 ± 0.71	1.558	0.119
COR (ng/ml)	204.31 ± 40.06	161.33 ± 27.83	34.076	< 0.001
BMI (kg/m^2^)	23.24 ± 3.76	22.01 ± 3.52	9.268	< 0.001

*SHS* suboptimal health status, *SBP* systolic blood pressure, *DBP* diastolic blood pressure, *GLU* plasma glucose, *TCH* total cholesterol, *TG* triglyceride, *HDLC* high-density lipoprotein cholesterol, *LDLC* low-density lipoprotein cholesterol, *COR* serum cortisol

*Case study four* — Association between ideal cardiovascular health metrics and suboptimal health status in a Chinese population

Another study explored the association between SHS and ideal cardiovascular health (CVH) metric scores. The study found higher ideal CVH metrics are associated with a lower prevalence of SHS, with subjects in the highest quartile of the ideal CVH metric summary score have a 57% reduced OR of having SHS compared to those in the lowest quartile [23]. The study found evaluation of SHS, combined with the analysis of seven cardiovascular health metrics as defined by the American Heart Association (smoking, physical activity, dietary intake, BMI, blood pressure, fasting blood glucose, and total cholesterol) allows for risk classification of CVD [23]. Four metrics (smoking, physical inactivity, poor dietary intake, and blood pressure) were significantly associated with the risk of SHS. Conversely, three metrics (ideal dietary intake, ideal physical activity, and never smoked or quit smoking > 12 months) were shown to be independent protective factors of SHS [23]. This demonstrates that health behaviour plays an important role in contributing to the association between SHS and risk of cardiovascular events. These findings suggest that increasing ideal CVH metric score is a new independent protection factor of SHS [23].

Taken together, these studies provide examples that detection of SHS effectively stratifies patients at risk of CVD [24]. Moreover, it can be proposed that intervention of SHS together with maintaining ideal CVH could be an effective preventive strategy for CVD from the perspectives of PPPM [23].

In conclusion, we demonstrated that assessment of SHS identifies persons at high versus low risk: avoiding under- and over-diagnosis with significant economic impacts to healthcare in the following ways; higher SHS scores are associated with T2DM and CVD [25, 29]; SHS evaluation, in conjunction with modifiable risk factor analysis, allows for risk stratification of patients for T2DM and CVD [23, 26]; increasing ideal CVH metrics score is a new independent protection factor of SHS [23]. These examples evidence SHS as the new standard in the global fight against chronic disease from the perspectives of PPPM. Effective intervention of SHS may be a cost-effective and time-efficient way for preventing chronic diseases, such as T2DM and CVD.

## The status quo of suboptimal health predictive diagnostic tools

### Health-related quality of life

The general concept of quality of life was initially considered a useful adjunct to traditional concepts of health and functional status [58]. It is a broad ranging concept comprising in a complex way the persons’ physical health, psychological state, level of independence, social interconnections and their environmental relationships [58]. The questionnaire developed by the World Health Organisation (WHO) is the WHO Quality of Life-100 (WHOQOL-100). It consists of 25 facets (4 items per facet; the total items = 100) in six dimensions: (1) physiology, (2) psychology, (3) independence, (4) social relations, (5) environment, and (6) spiritual support, religion, and personal belief [58]. The WHOQOL-100 was further revised for studies on health-related quality of life for a given group, such as WHOQOL-Brief and WHOQOL-Old [59]. These questionnaires have good psychometric properties such as reliability, validity and responsiveness and have been used to assess generic quality of life issues affected by all health problems [60].

The 36-item-short form health survey questionnaire (SF-36) is another popular instrument for evaluating health-related quality of life (61). It includes eight multiple-item with subscales to evaluate physical function, social functioning, role limitations due to physical problems and emotional problems, mental health, vitality, pain, and general health perception [62].

Health-related quality of life, by definition, covers a broader range of health-related status. Thus, specific tools are needed for the studies focused on the suboptimal health status.

### Suboptimal health status questionnaire-25

The suboptimal health status questionnaire-25 (SHSQ-25) has been developed for measuring SHS and validated in three major ethnic groups: African, Chinese and Caucasian. “SHSQ-25 includes 25 items covering five dimensions: fatigue, the cardiovascular system, the digestive tract, the immune system, and mental status” [63] (Table 1). SHSQ-25 questionnaire can be used in both health care and community settings to identify individuals who complain of poor health without a diagnosable condition [18, 31]. Each participant was asked to rate a statement on a 5-point Likert-type scale, based on how often they had experienced a particular complaint in the previous 3 months: (1) never or almost never, (2) occasionally, (3) often, (4) very often, and (5) always. The raw scores of 1 to 5 on the SHSQ-25were recoded as 0–4 [27]. Table 3 presents the specialised questionnaire SHSQ-25.

**Table 3:** Suboptimal Health Status Questionnaire-25 (SHSQ-25); these questions inquire about health events occurring during the past 3 months. Every question is required to be marked with an “x” in the appropriate box; then, the scores are totalled for a SHS score (Yang et al. 2009; Wang and Yan 2012)

In the preceding 3 months, how often was it that you (your)…	1	2	3	4	5	Score
	Never or almost never	Occasionally	Often	Very often	Always	
1. Were exhausted without greatly increasing your physical activity?						
2. Experienced fatigue that could not be substantially alleviated by rest?						
3. Were lethargic when working?						
4. Suffered from headaches?						
5. Suffered from dizziness?						
6. Eyes ached or were tired?						
7. Suffered from a sore throat?						
8. Muscles or joints felt stiff?						
9. Have pain in your shoulder/neck/waist?						
10. Have a heavy feeling in your legs when walking?						
11. Felt out of breath while sitting still?						
12. Suffered from chest congestion?						
13. Were bothered by heart palpitations?						
14. Appetite was poor?						
15. Suffered from heartburn?						
16. Suffered from nausea?						
17. Could not tolerate cold environments?						
18. Had difficulty falling asleep?						
19. Had trouble with waking up during night? i.e., kept waking up at night						
20. Had trouble with your short-term memory?						
21. Could not respond quickly?						
22. Had difficulty concentrating?						
23. Were distracted for no reason?						
24. Felt nervous or jittery?						
25. Caught a cold in the past 3 months?						
Total						

The SHSQ-25 is short and easy to complete and, therefore, an instrument suitable for use in both large-scale studies of the general population and routine health survey [20]. To date, SHSQ-25 as a practical SHS survey tool has been verified in various populations and used as an auxiliary tool to screen high-risk groups. For example, a SHSQ-25 survey showed that SHS was an independent risk factor for T2DM [25] and it can also be used as an auxiliary screening tool to explore the comprehensive risk factors of cardiovascular disease [24].

Therefore SHSQ-25 provides a window of opportunity for early detection and intervention of diseases, and helps to reduce the burden of diseases [21]. Except SHSQ-25, there are other SHS measures tools available in Chinese language, which are mainly used in Traditional Chinese Medicine (TCM) practices, e.g., Sub-health Self-rating Scale (SSS) has been invented for student SHS measure [64]. SSS consists of three symptom dimensions, with ten factors supported by 58 items.

### Objective* measures for SHS*

Biochemical and molecular biological tests are of great significance for evaluating health status and predicting disease progression. Due to the complexity and diversity of SHS symptoms, there is no accurate and unified index system for biochemical assessment of suboptimal health, but some achievements have been obtained in related studies mainly involving laboratory examination and related animal experiments [65].

#### General biological and biochemical indexes

Systolic blood pressure, diastolic blood pressure, total cholesterol, and high-density lipoprotein cholesterol were fund to be associated with SHS [18]. In addition, plasma glucose was associated with SHS in men, and triglyceride in women respectively [18]. The SHS group had higher serum cortisol than that of health groups and there was a significant linear correlation between the SHS scores and serum cortisol level [18].

Combined with a stress test tool, Copenhagen Psychosocial Questionnaire (COPSOQ), and further integrated with the analysis of the expression of glucocorticoid receptor mRNA (GR mRNA), SHSQ-25 proved to be a tool to assess chronic psychosocial stress. Plasma cortisol and glucocorticoid receptors are important physiological mediators of psychological stress [66]. Plasma cortisol was significantly increased in subjects with high SHS scores and the expression of GR mRNA in peripheral blood monocytes was significantly decreased [66]. Another study also confirmed the unique value of plasma cortisol in the assessment of SHS [67].

A study in Ghanaian women showed that oxidative stress is closely related to SHS, and endothelial dysfunction, indicating that a complication of oxidative stress, may also have a certain correlation with SHS [32]. Another population based study in Russia, which combined measure of endothelial dysfunction with the SHSQ-25 to assess cardiovascular suboptimal health, demonstrated a significant association between endothelial dysfunction and suboptimal health [24]. The group with high SHS score showed the significantly higher levels of systolic blood pressures (SBP), diastolic blood pressures (DBP), body mass index (BMI), smoking index, total cholesterol (TCH), triglycerides (TG), and low-density lipoprotein (LDL) cholesterol [24]. Integration of suboptimal health status and endothelial dysfunction provides a novel tool to allow people to get a more holistic picture of both subjective and objective health measures and also can be applied to routine screening for risks of cardiovascular diseases [24]. Recently the SHSQ-25 tool has been adapted as African version of SHSQ-25 from the African context [68].

#### Molecular biological measures

As SHS is the transition stage from health to disease, the study on the quantitative change from the perspectives of proteomics, glycogenomics, metabolomics and telomere length might provide another point of view for SHS pathogenesis.

Proteomic studies showed that many SHS people have immune abnormalities, involved in cytokines-mediated immune responses with inflammatory consequence. A controlled study in Japanese population found that mRNA levels and production of transforming growth factor-beta1 (TGF-β1) were significantly reduced in people with chronic fatigue syndrome (CFS) [69]. The lack of cytokine in CFS may lead to myalgia and muscle fatigue.

**Fig. 3:**
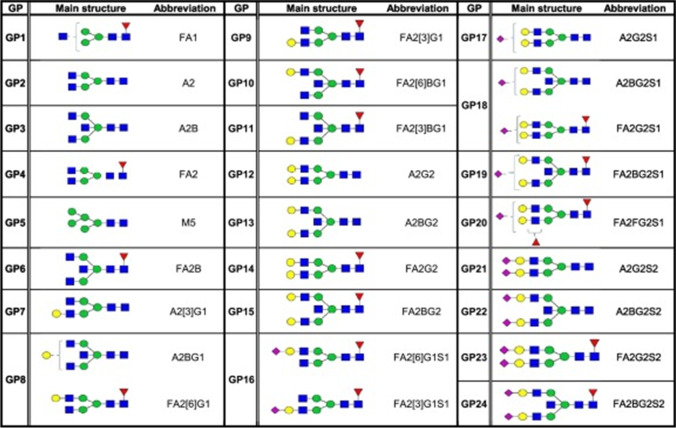
The 24 IgG glycan peaks as measured with ultra-performance liquid chromatography (71). Shown are the structure of the main IgG glycan component(s) per peak and their structure abbreviation. Structure abbreviations: F, α-1,6-linked core fucose; A, number of antenna's attached to the core sequence (existing of two N-Acetylglucosamine (GlcNAc) and three mannose residues); B, bisecting GlcNac β1–4 linked to β1–3 mannose; M, number of mannose residues; G, number of β1–4 linked galactoses; [3] G1, galactose on the antenna of the α1–3 linked mannose; [6] G1, galactose on the antenna of the α1–6 linked mannose; S, sialic acid linked to galactose. Structural schemes are defined as follows: blue square, GlcNac; green circle, mannose; red triangle, core fucose; yellow circle, galactose; and purple rhomb, sialic acid

SHS is a contributing risk factor for metabolic syndrome. N-glycan is associated with metabolic syndrome, according to suboptimal health and glycosomics studies conducted in Ghanaian population [70]. Four glycan peaks (GPs), GP8 (FA2[6]G1), GP18 (FA2G2S1), GP21(FA2G2S2), and GP34 (A4G4S[3,3,6,]3), were found to predict sub-health and metabolic syndrome status (70). Figure 3 highlights glycomics as the SHS diagnostic tool.

Metabolomics studies have demonstrated that chronic fatigue syndrome is a highly coordinated hypometabolic response, identifying metabolomics as a powerful tool for identifying chemical differences between health and disease [71]. A combination of four metabolic biomarkers (sphingosine, pregnanolone, taurolithocholate sulfate, cervonyl carnitine) is able to distinguish SHS individuals from the controls with a sensitivity of 94%, a specificity of 90%, and an area under the receiver operating characteristic curve of 0.977 [72]. SHS-related metabolic disturbances can be detected at the early stage and SHS-related metabolites creates a window of opportunity for chronic diseases management from the perspective of PPPM [72].

In addition, there were studies reporting that women had a higher collagen/non-myofibromyin turnover in the amino acid composition of urine, indicating diverse characteristics of the urinary excretion of amino acids in humans and suggesting the use of amino acid supplementation to reduce fatigue and suboptimal health in adults [73].

Besides of these molecular biological measures, a study measuring relative telomere length (RTL) found that SHS was significantly associated with short RTL, indicating combination of subjective (SHS) and objective (RTL) measures is a novel tool for accelerated ageing investigation [63].

### The outlook of SHS diagnostic tools

Previous* studies* have proved that SHSQ-25 has a wide range of applicability. It is an inevitable task to translate SHSQ-25 into other diverse languages. Currently, there are three languages versions of SHSQ-25, Chinese, English and Russian. We need more effort to translate SHSQ-25 into more local languages to apply it amongst different ethnic groups.

*In *animal* models for SHS mechanism study*, although some animals, such as rat and mice, have been experimented to simulate the physiological and psychological negative status of human SHS, they cannot be completely equivalent to human [74, 75]. Animal study will improve our knowledge on SHS mechanism.

*The *existing* SHS assessment criteria and the thresholds* are based on the cut off values which do not meet the ranges for clinical disease diagnosis, and the range value is narrow, so a single or a few indicators are often difficult to truly reflect the SHS of an individual. In this regard, the reliability and validity of diagnosis may be greatly improved by using large sample investigation or experiment, screening the combination of physiological and biochemical indicators, and constructing the detection index spectrum of different subtypes of SHS.

The research on molecular biology of SHS has made achievement, but it has been mainly limited to laboratory detection, lacking convenient, fast, sensitive, efficient and low-cost practical detection tools [66, 67]. The development of high-through-put, standardised and practical biological detection chips or kits will be the main direction of diagnosis or screening. For example, N-glycans are crucial for intra/extracellular interaction or signalling, cell adhesion, translocation, cell–cell or molecule-cell interaction, inflammation and immune function and they are dynamic biomarkers for monitoring physiological conditions. The advanced glycomics detection measurement will become a powerful objective measure to evaluate SHS [76, 77].

The future SHS research must follow a comprehensive, large sample, qualitative and quantitative paradigm. This will produce a huge amount of data, coupled with the massive bioinformatics data generated by molecular biology technology, and the demand for data processing and analysis will become more and more prominent. In future, more effective data analysis and mining measures and artificial intelligence will not be absent.

## Suboptimal health status, risk factors and risk group

Suboptimal health status (SHS) has several adverse outcomes due to physical, psychological, and social stress of an individual [21]. However, its main underlying mechanism is still unclear [78]. As a result of physical and psychological changes, the overall coordination of the body system becomes imbalance and dysfunction (the nervous system, endocrine system, and immune system), these lead to decline of physiological, psychological and social functions; however, the body has not yet moved to the disease stage [20, 78, 79].

“People with SHS face different chronic conditions, such as chronic fatigue, headache, dizziness, depression, anxiety, loss of appetite, insomnia and functional disorders” [27]. Similarly, several organ systems, non-specific pain, functional disorders of different organ systems (the digestive system, cardiovascular system, respiratory system, urinary system) are associated with short-term or long-term adverse health outcomes [80]. For example, SHS plays a vital role in impairing the quality of life, resulting in frequent hospital visits and significant medical expenses. Still, no obvious symptoms of any particular disease appear, making the victims frustrated thus disabling their normal life [27, 78].

The prevalence of SHS is high in China [79], and consistently it has been growing in other countries. SHS's symptoms might be underestimated everywhere, and many individuals do not know about suffering from SHS [27]. For example, a study conducted in China with 6000 healthy people found that about 72% of participants had SHS [79]. Therefore, many scientists worldwide have focused on age, gender, occupation, geographic region, and behaviour of persons at high risk of having SHS in recent days. A personal health monitoring mechanism is required to prevent SHS and chronic disease. To focus on the above issue, recently introduced PPPM (predictive, preventive, and personalised medicine), a new paradigm of public health services technique intensively expanded worldwide [81, 82].

### Relationship between chronic diseases and SHS

According to WHO [83], non-communicable diseases (NCDs), also called chronic diseases, kills 41 million people per year globally. Likewise, more than 15 million people die each year from NCD age between 30 and 69 years old (a productive age group), and these premature deaths occur mainly in low- and middle-income countries (LMICs), which is 77% of all NCD death (84). In addition, alone cardiovascular diseases (heart attacks and stroke) kill about 17.9 million people, followed by 9.3% cancer, 4.1 million by respiratory conditions such as chronic obstructive pulmonary diseases (COPD), and 1.5 million people with diabetes per year around the world. Notably, these four diseases record more than 80% of premature deaths by NCD [83].

Curiously, WHO again stated that tobacco use, physical inactivity, alcohol use, and unhealthy diets are the risk factors to increase NCD deaths. Similarly, children, adults, and the older adults are more vulnerable to the risk factors contributing to NCDs, that may be due to above risk factors [83].

On the other hand, SHS is regarded as a subclinical reversible stage of chronic diseases and has been found a potential risk factor for chronic conditions, e.g., diabetes mellitus, cardiovascular and stroke, including several cancers and metabolic diseases [20, 80]. Many evidences show that an individual’s modifiable behaviour, such as smoking, insufficient physical activities, unfit eating habits, and excessive alcohol intake, are the risk factors for NCDs [83, 85, 86]. About 7.2 million people deaths each year by tobacco use, following 4.1 million deaths by high salt intake, 3.3 million people by alcohol use associated with cancer, and 1.6 million deaths due to physical exercise [84]. Therefore, WHO has made a target to reduce premature deaths from NCDs through a sustainable development agenda by 2030 [87].

There are numerous risk factors for the occurrence of chronic diseases, e.g., socio-economic status, education level, daily physical exercise, salt intake, blood pressure, cardiovascular markers, triglycerides, cholesterol, alcohol use, and a poor eating habit [21, 78, 86]. Many studies suggested that cigarette use, physical inactivity, unbalanced diet, insufficient sleep, and uncontrolled blood pressure are directly associated with SHS risk [27, 29, 78, 80, 88]. Similarly, these risk factors are associated with a lifestyle or behaviour of a person, and these unhealthy lifestyles or behaviour play a significant role in increasing the SHS [78, 88, 89].

### Lifestyle* factors and SHS*

A study found that lifestyle factors have a stronger indirect association with physical, mental, and social SHS by health consciousness than direct physical, mental, and social SHS associations [79]. This study also established that health consciousness has a strong direct relationship with physical, mental, and social SHS [90]. However, many studies have been recognised that lifestyle factors are important risk factors for SHS [27, 91]; those include smoke habits, alcohol use, skipping breakfast, dietary inconsistency, exercise deficiency, and sleep problems [27, 91, 92]. Consistently, SHS is associated with risk factors of chronic diseases and subsidised to developing these diseases [93] Therefore, everyone can be prevented from chronic diseases by modifying their poor lifestyle behaviours.

A healthy lifestyle includes a multidimensional pattern of self-initiated perceptions and activities to maintain and improve individual’s health and wellbeing [91, 93]. These kinds of perceptions and activities can reduce the occurrence of the disease, decrease the death rate and contribute to improving health status. On the other hand, risky health behaviours play a significant role in increasing the risk for injury and disease conditions [85], such as tobacco and alcohol use, a poor diet habit, and lack of body activities, which lead to various chronic diseases. The WHO has also emphasised that unhealthy behaviour and lifestyle are important factors amongst the top ten causes of death worldwide [94–97].

### Adolescent* period and SHS*

Adolescent age (10 to 24 years) is a crucial age, transitioning between childhood and adulthood [98]. This period is characterised by pubertal maturation, the transition towards the mature for adulthood’s social roles and developing independence [99]. Therefore, it is also called a time of “Storm and Stress” [100, 101]. Especially, the preadolescent period poses a unique opportunity to affect family health behaviours before the child becomes more peer-focused. In addition, to develop confidence ability to be physically active (self-efficacy), the idea about the consequences of being physically active (beliefs) and influences of family and friends on physical activities (social influences) affect the physical activity of adolescents [102]. The adolescent is an intensified influencing time to expose with peer group and adopt unhealthy behaviour such as alcohol use, smoking habit, unhealthy diet etc. [99]. These consequences affect both current and future health status; however, the adolescent period also provides considerable opportunity for behaviour to be shaped in a positive way that may improve long-term health outcomes [99].

Epidemiological data showed that risk behaviours are the leading cause of adolescent morbidity and mortality globally [95]. The risk behaviour reaches a peak in adolescence, signifying that this period demonstrates a high propensity or inherent tendency to take risks [97]. Because of their high-risk taking tendency, they often suffer accidents or injuries [103]. This type of risk-taking behaviour during adolescence always puts them at risk [101].

During the adolescent period, several changes occur, such as physical development, reproductive health development, mental health, social and emotional changes. Lifestyle behaviours during youth are the foremost important period of life. That is why adulthood is an important age for forming healthy behaviour or poor behaviour, and those factors are associated with an increase in the risk of chronic diseases [104]. Though, unhealthy practices and behaviour, along with poor lifestyles of young ages, may be a pathway to a whole lifespan, determining the increased cause of health risk in old age [91]. Unhealthy behaviour during adolescence can play a vital role in developing health consequences in later life [105].

Likewise, students’ risky behaviour during their college or university life is also a crucial part of increasing diseases appearance in the second part of their life [106]. The study showed that SHS was high in high school students, where the detection rate of physical SHS and Psychological SHS was elevated [78]. The physical and mental development during adolescent age may be an ideal time to know the health information. This information would be a piece of evidence to understand the level of health status. Thus, the health assessment information during the college or university period can be the more convincing evidence to prevent chronic disease for the future age [78].

It has been acknowledged that SHS can be prevented if it is diagnosed in the early stage and starts timely management. Therefore, a new treatment paradigm or mechanism is originated to enable people for early intervention of disease in terms of PPPM [80–82].

## Individualised preventive measures

The natural history of a disease stages includes: underlying, susceptible, subclinical, clinical, and recovery/disability/death [107]. Correspondingly, according to the WHO guidelines, health prevention have been grouped into primary, secondary, and tertiary prevention [108]. The combination of these preventive measures can prevent the onset of disease through risk reduction, and also the downstream complications of manifested diseases [107].

### Individualised disease prevention

*Primary prevention* refers to measures aimed to avoid the manifestation of a disease at both individual and population levels [109]; therefore, its target population is the healthy individuals [107, 110]. It commonly institutes actions that prevent from exposures to hazards, increase immunity, mitigate disease risk, alter unhealthy behaviours, or supply nutrition and food to prevent disease progress from a susceptible individual to the subclinical stage of disease [108].

*Secondary prevention* emphasises early detection, which improves the chances for positive health outcomes [108]. Secondary prevention targets healthy-appearing individuals in the subclinical stage of the disease [107]. The subclinical disease is regarded as the initial period of a disease with no recognisable symptoms [111]. The main form of secondary prevention is evidence-based screening programs implemented at the early stage of a disease [109, 110].

*Tertiary prevention* is administered in symptomatic patients [108]. It aims to reduce effects of the ongoing disease on individuals, commonly by treating disease, providing rehabilitation, eliminating disability, screening complications, halting disease progression, restoring function, minimising suffering, and maximising potential years of quality life [112–114]. However, the intervention is by no means a cure, and close follow-up of chronic disease are required including supervising medications taking, monitoring changes, and supporting patients to maximise the capacity to live independently in daily life is an essential element of public health [114].

*Individualised prevention*, which may also be referred to as personalised prevention, predictive, stratified prevention, is an attempt to refocus attention to preventing disease at the level of the individual [115, 116]. Biological variation plays a major role in individualised prevention because the most accurate and effective prevention for an individual is based on individuals’ health status, his or her unique genetic, environmental and behavioural factors [116, 117]. Form the perspectives of predictive, preventive and personalised application, the current clinical applications involve the use of subjective and objective measurements. Subjective measurements are dependent on observers’ opinions, feelings, and general impressions, and self-report questionnaires are the main types of assessment approach. For example, Suboptimal Health Status Questionnaire-25 (SHSQ-25), a reliable, valid, and robust and targeted health measure tool, is applied to assess Suboptimal Health Status (SHS). “The concept of SHS, an intermediate and reversible physical state between health and disease, illuminate the viewpoint that non-communicable chronic diseases can be effectively predicted and prevented before the occurrence of a clinical manifestation of severe pathologies” [1, 21, 118]. Objective measurements are impartial, usually quantifiable outcomes recorded, and one of most common approach to quantifying objective outcomes is the measures of biomarkers, such as C-reactive protein (CRP), interleukin-6 (IL-6), tumour necrosis factor alpha (TNF-α), DNA methylation, and immunoglobulin G (IgG) N-glycan profiling. Glycosylation, a common co- and post-translational modification of IgGs, mediate anti-inflammatory and pro-inflammatory responses through affecting the IgGs’ folding, structure, stability and effector functions. Identifying biological mediators of SHS will not only help with effective interventions to reverse SHS, but clarify the mechanisms between SHS and SHS-related chronic disease. Glycosylation of IgG is an important regulator of the immune responses and changes in glycan structures may provide information about the mechanisms of SHS and the etiological link between SHS and chronic disease, such as metabolic syndrome (MetS) [119], cancer [120], Parkinson’s disease [121, 122], systemic lupus erythematosus (SLE) [123], rheumatoid arthritis (RA) [124], dyslipidemia [125], hypertension [126], cardiovascular diseases [24], type 2 diabetes mellitus (T2DM) [127], stroke [126], and ageing [128], that can then provide strategies for the a reversal of SHS.

*In practical terms*, the individualised prevention not only aims to replace existing classic, population or community-based public health efforts, but to ensure individuals can access the right prevention with accurate dose and at the proper time to optimise the benefit-risk ratio of all interventions maximising efficacy and safety [129]. Hence, both primary and secondary prevention can be precisely applied, providing cost-effective and tailored preventive strategies for the reversal of SHS [117, 129].

### Preventions for established objective measurement of SHS

From the perspective of PPPM, the concept of SHS reflects “the viewpoint that chronic diseases can be effectively predicted and prevented before the occurrence of a clinical manifestation of severe pathologies” [11]. Consequently, the early identification of SHS has the potential to predict and prevent NCDs at the early stages, and effective prevention on SHS may be a cost-effective way for preventing NCDs [118]. In order to fast and cost-effectively evaluate SHS, and in line with PPPM, we developed “the innovative, reliable, valid and robust health measure tool, Suboptimal Health Status Questionnaire-25 (SHSQ-25)” [27].

**Fig. 4:**
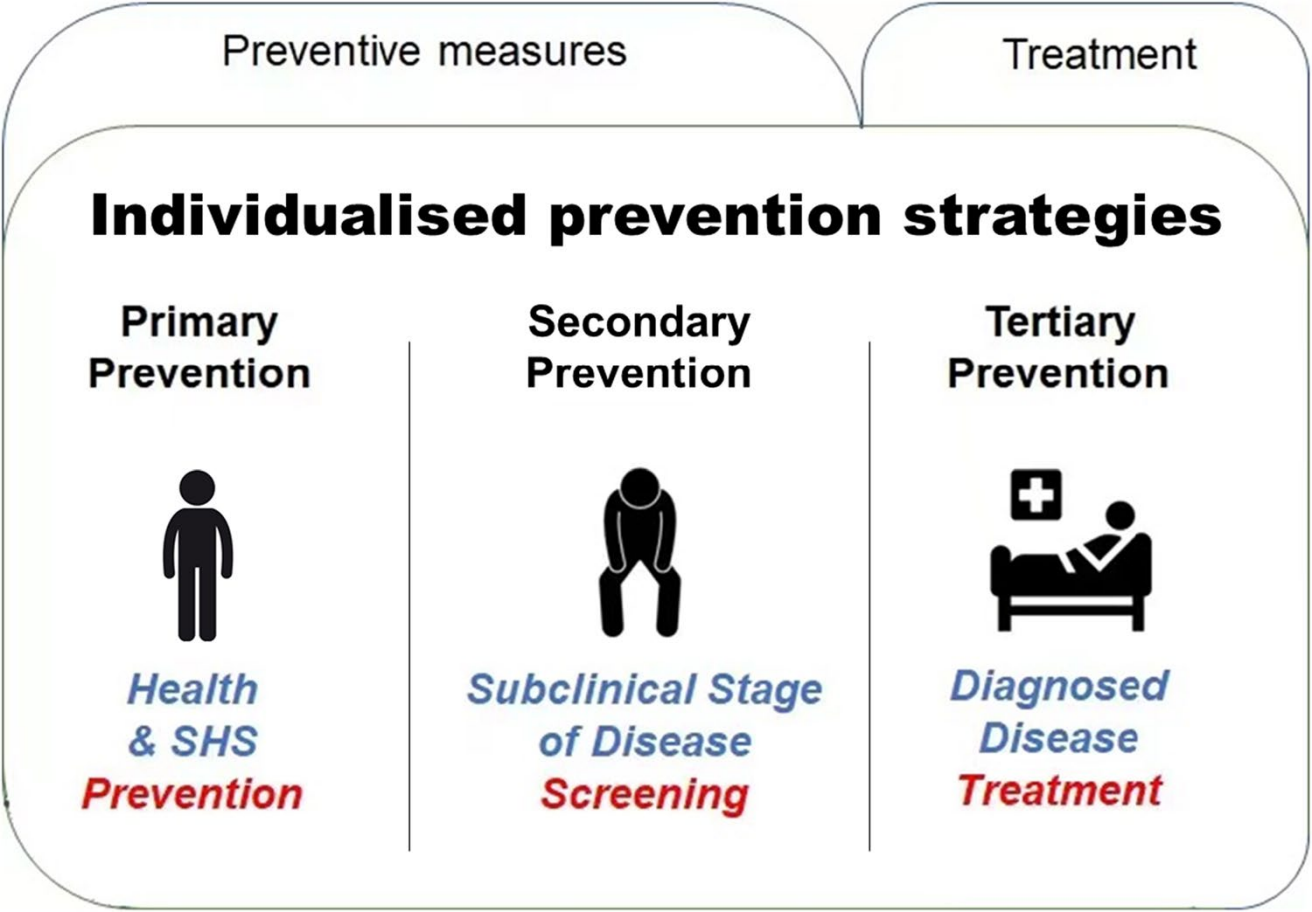
Individualised prevention strategies. Primary prevention includes individuals with no disease who receive preventive measures. Secondary prevention is screening to detect subclinical stages of diseases. Tertiary prevention is management of disease. SHS, suboptimal health status

The key principle of disease prevention (Fig. 4) is to apply PPPM strategies to prevent the development of the disease at the onset of SHS. Ideally, intervention should occur at the point where there are relevant symptoms deviating away from health, yet do not meet the standard diagnostic criteria of a diagnosable disease. To prevent SHS, we should pay attention not only to the physical health status when non-recognisable symptoms of diseases approach, but also to the emotional and mental status. It is necessary to apply preventive strategies by the person-tailored intervention approaches to provide cost-effective and tailored preventive measures based on the integrative concept PPPM. Here, we present case studies to prove the effectiveness of preventive measures on SHS.

*Case study one:* Intervention effect of *Baduanjin* exercise in people with SHS

**Fig. 5:**
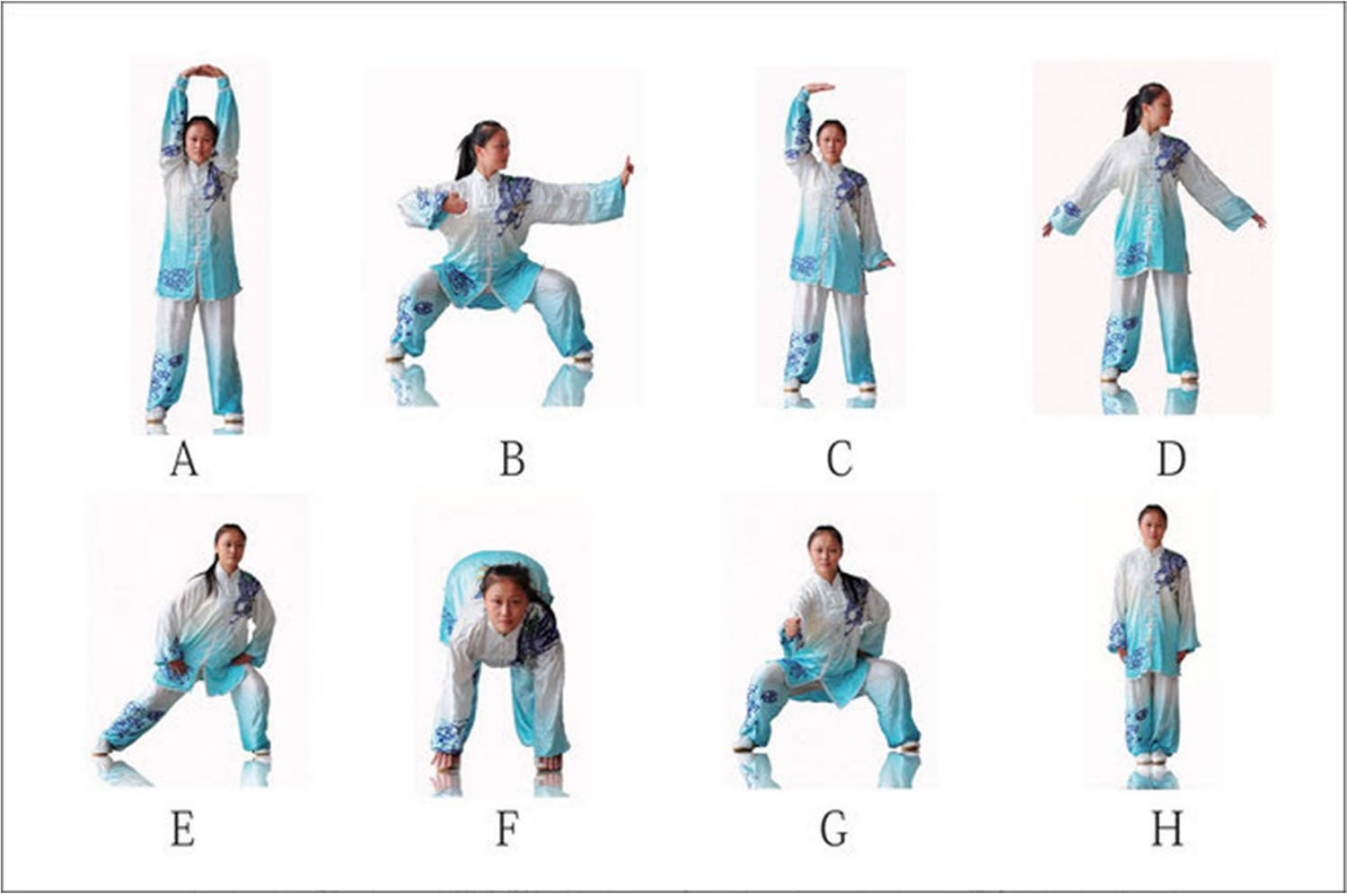
Standardised *Baduanjin* exercise by the Chinese Health *Qigong* Association (134). Each of the displayed movement brings certain function-enhancing benefits to particular parts of the body. **A** Prop up the sky with hands to regulate the triple energiser. **B** Draw a bow on both sides like shooting a vulture. **C** Raise single arm to regulate the spleen and stomach. **D** Look back to treat five strains and seven impairments. **E** Sway head and buttocks to expel heart fire. **F** Pull toes with both hands to reinforce the kidney and waist. **G** Clench fists and look with eyes wide open to enhance strength and stamina. **H** Rise and fall on tiptoes to dispel all diseases

Growing evidence continues to prove that regular exercise or physical activity can contribute to improve physical and psychological health conditions [130]. Moreover, traditional Chinese exercises, such as *Baduanjin* exercise, are effective exercise programs for enhancing health promotion in body composition, physical fitness, and mental health [131–133]. *Baduanjin* exercise consists of eight separate, delicate, and smooth exercise movements: each movement brings certain function-enhancing benefits to particular parts or organs in the body (Fig. 5) [134].

**Fig. 6:**
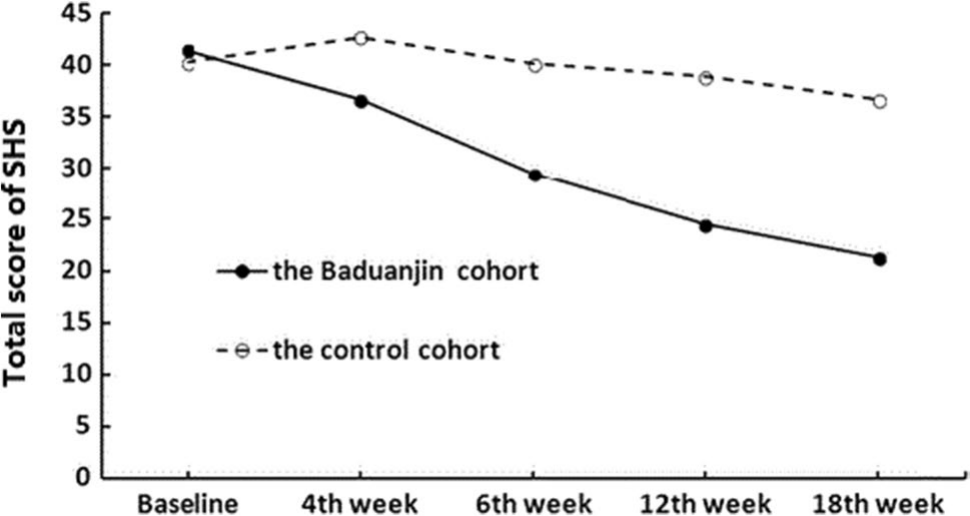
Trends in changes in the scores for SHS [50]. SHS, suboptimal health status

To evaluate the effect of *Baduanjin* exercise on fatigue in people with SHS, a clinical trial was conducted. A total of 131 participants was recruited and allocated into the *Baduanjin* group (*n* = 64) and the control group (*n* = 67) [135]. After intervention, participants were followed up for 12 weeks to assess the sustained effect of *Baduanjin* exercise on SHS. Statistical analyses were conducted to compare the SHS score at different time points, including baseline, 4th and 6th week after the intervention, and 12th and 18th week during the post-intervention follow-ups. The results showed SHS scores were significantly decreased (*P* < 0.05) (see Fig. 6).

*Case study two:* Effectiveness of mild moxibustion for SHS in pre- and post-menopausal women

Due to dysregulated endocrine functions, peri-menopausal women often suffer from SHS with discomfort symptoms, including fatigue, insomnia, hot flushes, irritability, mental confusion, headache, and difficulty concentrating [136]. Moxibustion, a form of heat therapy in which dried plant materials called “moxa” are burned on acupoints, has been reported as a clinical treatment method and for self-care at home amongst Chinese and other Asian people [137]. To investigate the efficacy and safety of using mild moxibustion for treating SHS in peri-menopausal women, a randomised controlled trial (RCT) was conducted in 60 participants randomly assigned to the moxibustion and control groups. The moxibustion group received mild moxibustion and the control group gained vitamin E soft capsules [138]. The results showed SHS significantly improved in the moxibustion group compared with the control group (*P* < 0.01). Compared to post-menopausal women, pre-menopausal women showed more prominent improvements in SHS (*P* < 0.05). In addition, there was a significantly higher estradiol (E2) levels in the moxibustion group than the control group after treatment (*P* < 0.01), and pre-menopausal women had higher E2 levels in the moxibustion group when compared to post-menopausal women (*P* < 0.01). In comparisons of pre- and post-menopausal women, pre-menopausal women had significantly higher levels of E2 (*P* < 0.01), prolactin (PBL; *P* < 0.05) and progesterone (*P* < 0.01), while levels of luteinising hormone (LH) and follicle-stimulating hormone (FSH) were significantly higher in post-menopausal women (*P* < 0.01).

*Case study three:* Effects of pulsatile cupping on body pain and quality of life in people with SHS

Cupping therapy is an ancient medical technique, and it applies heated cups on the skin to remove stagnation and stimulate the flow of “*Qi*” [139]. Due to its non-invasive simple operation and rapid effects, cupping has been broadly used in China and other Asian population [140]. In clinical practice, cupping has been reported as a main clinical treatment method to relief SHS and increase patients’ general feeling of wellbeing [141].

**Fig. 7:**
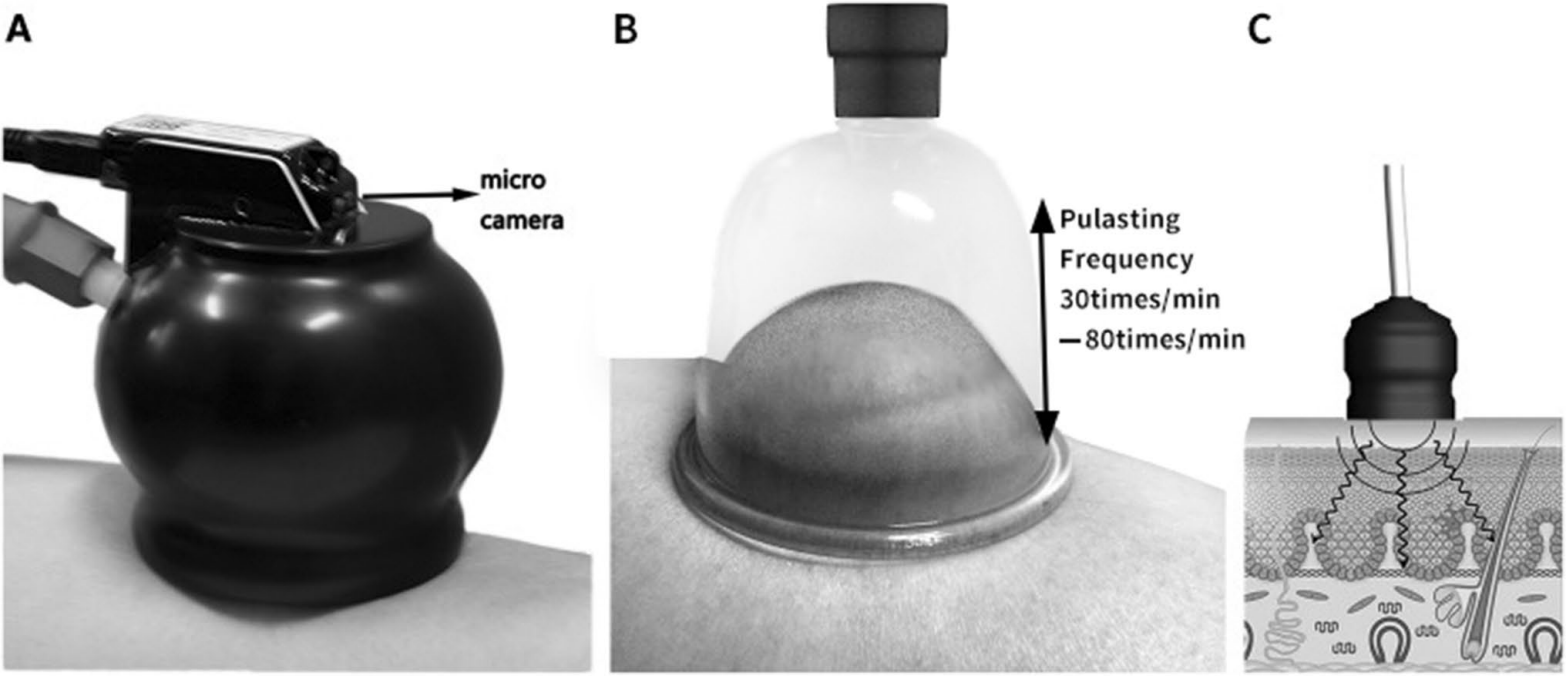
Pulsating cupping devices, the figure is adapted from (142). **A** One tube connects with a Bian-stone cup. In the bottom of the Bian-stone cup, a micro-camera is set and connected wirelessly to a computer to monitor colour changes in the cup. **B** One tube connects with an acrylic cup to show the vibrations at various frequencies. **C** Schematic diagram of the device shows complex stimulation (vacuum and vibration) performed on the local tissues of the skin. min, minutes

A four-arm, randomised clinical trial was conducted to investigate effects of the pulsating frequency of pneumatic pulsatile cupping, compared with traditional cupping, on body pain and quality of life in people with SHS [142]. This study recruited 96 SHS participants randomised to low-frequency (LF) or high-frequency (HF) pulsating cupping (see Fig. 7), traditional cupping (TC) or wait-list (WL) groups.

After treatment, the symptom of pain was significantly reduced in LF and HF groups when compared to TC group (*P*^LF^ = 0.048; *P*^HF^ = 0.004), respectively; and the quality of life was significantly improved in LF and HF groups (*P*^LF^ = 0.046; *P*^HF^ = 0.004). Moreover, in comparisons of the WL group, the symptom of pain significantly reduced (both *P* < 0.0001) whilst the quality of life significantly improved (both *P* < 0.0001) in LF and HF groups. However, there was no significant difference between LF and HF groups (*P* > 0.05).

### Preventions-established objective measurement of SHS

When health deteriorates (i.e., in SHS) the molecular processes are already perturbed in a measurable way, but there is still no condition that meets the clinical diagnostic criteria of disease. At molecular level, immunoglobulin G (IgG) glycosylation plays a critical role in inflammation process (70); thus, glycans are conceived as one of the ideal objective biomarkers for measuring SHS.

**Fig. 8:**
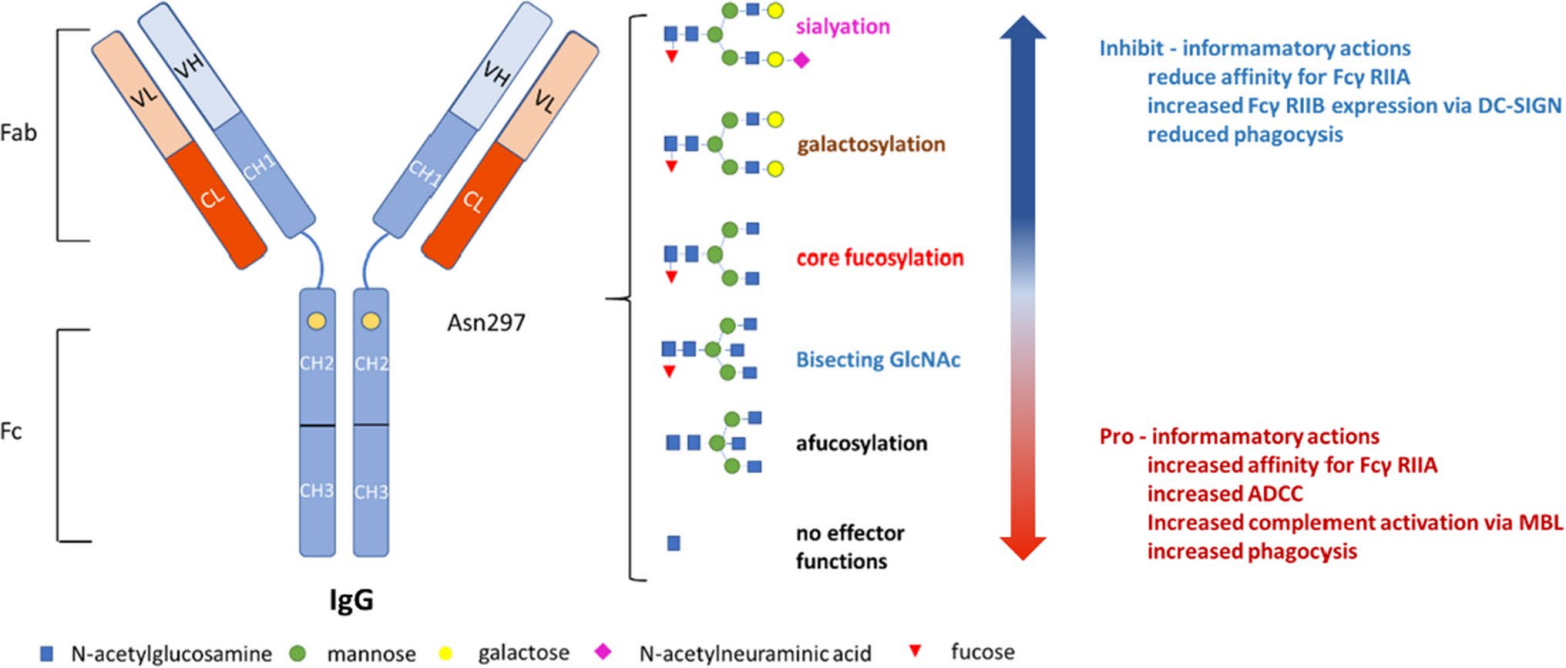
Immunoglobulin G effector functions are modulated by Fc-bound N-glycans, the figure is adapted from. GlcNAc, N-acetylglucosamine; ADCC, antibody-dependent cellular cytotoxicity; FcyRIIB, Fc gamma receptor type IIB; MBL, mannose binding lectin

The IgG, linking the innate and adaptive branches of the immune system, is one of the most extensively characterised glycoproteins overall [143]. The effector’s functions of IgG are modulated via fragment crystallisable (Fc) N-glycans [144]. In addition to directly affecting conformation of the fragment crystallisable (Fc) region, conserved N-glycans attached to the constant heavy 2 (CH2) domain of the IgG Fc region also mediate downstream immune responses (i.e., anti-inflammatory or pro-inflammatory responses; see Fig. 8) [121, 145]. Additionally, variations to the IgG N-glycans can elicit ageing at the molecular level through a process generalised as inflammageing [146]. Since IgG N-glycans are valuable biomarkers for SHS identification and create a window opportunity for PPPM of SHS, IgG N-glycans can be adapted as the molecular parameter for individualised monitoring and prevention of SHS.

The epidemics of obesity, physical inactivity and unhealthy diet are driving an increase in SHS that is a new challenge for both population and individual health. The increase in costs with regards to the management of non-communicable diseases is burdening healthcare budgets under increasing strain. Changing the focus towards SHS prevention is the only sustainable approach to this problem. Since IgG N-glycans can be adapted as a biomarker of SHS and its compositions can be modified through interventions, it is possible to monitor effects of beneficial interventions. This enables individualised management of lifestyles, thereby minimising the risk of SHS from the perspectives of PPPM.

*Case study one:* Intense physical exercise induces an anti-inflammatory change in IgG N-glycosylation profile

To explore effects of intense physical exercise on the IgG N-glycome composition, a longitudinal intervention study was conducted with 29 males. Samples were measured in three time points: pre-intervention, at the peak of the training program intensity (EXC), and one-month post-intervention (recovery period, REC). After the analysis of IgG N-glycosylation, results from the baseline, EXC, and REC time points were compared. The following derived glycan traits were analysed: agalactosylation, monogalactosylation, digalactosylation, monosialylation, disialylation, bisection, and core fucosylation. Estimated effects of exercise showed significant differences in IgG N-glycans in the REC time point compared to the baseline (Table 4): agalactosylated N-glycans decreased (− 0.8080, *P* = 0.0473), and both digalactosylated and monosialylated N-glycans increased (0.9949, *P* = 0.0473 and 0.5270, *P* = 0.0339, respectively) [147].

**Table 4:** Estimated effects of training intervention on levels of individual derived IgG N-glycan traits, and their respective unadjusted and adjusted *P*-values (147)

Structural feature	Estimate EXC	*P*-value EXC	Adj. *P*-value EXC	Estimate REC	*P*-value REC	Adj. *P*-value REC
Agalactosylation	− 0.2009	0.5394	0.6152	− 0.8080	0.0190	0.0473
Monogalactosylation	− 0.1040	0.5388	0.6152	− 0.1710	0.3149	0.4409
Digalactosylation	0.3049	0.4554	0.6152	0.9950	0.0203	0.0473
Monosialylation	0.2688	0.1283	0.6152	0.5270	0.0048	0.0339
Disialylation	0.1430	0.2206	0.6152	0.2465	0.0399	0.0698
Bisection	0.0901	0.5807	0.6152	0.1095	0.5030	0.5868
Core fucosylation	− 0.0373	0.6152	0.6152	− 0.0339	0.6479	0.6479

The exercise effect coefficients are calculated relative to the baseline. Significant results are shown in bold. *EXC* exercise intervention, *Adj.* adjusted, *REC* recovery period. Decreased agalactosylated N-glycans and rise in digalactosylated and monosialylated N-glycans attached to IgG suggest intense physical exercise indeed induced anti-inflammatory effects

*Case study two:* Effects of estradiol on biological age measured using the glycan age index

To evaluate the effects of ovarian sex hormone suppression followed by estradiol supplementation on biological age (measured by the glycan age), a double-blinded randomised controlled trial (RCT) was conducted based on a parental trial [148]. A total of 36 healthy young women were included in this study. All underwent suppression of ovarian gonadal hormones with gonadotropin releasing hormone agonist therapy (GnRHAG) for a period of five months. Meanwhile, participants were randomly allocated into estradiol (E2) intervention (GnRHAG + E2, *n* = 15) and placebo control group (GnRHAG + PL, *n* = 21). Inclusion and exclusion criteria, as well as protocol of the study can be obtained from the parent trial [149].

**Fig. 9:**
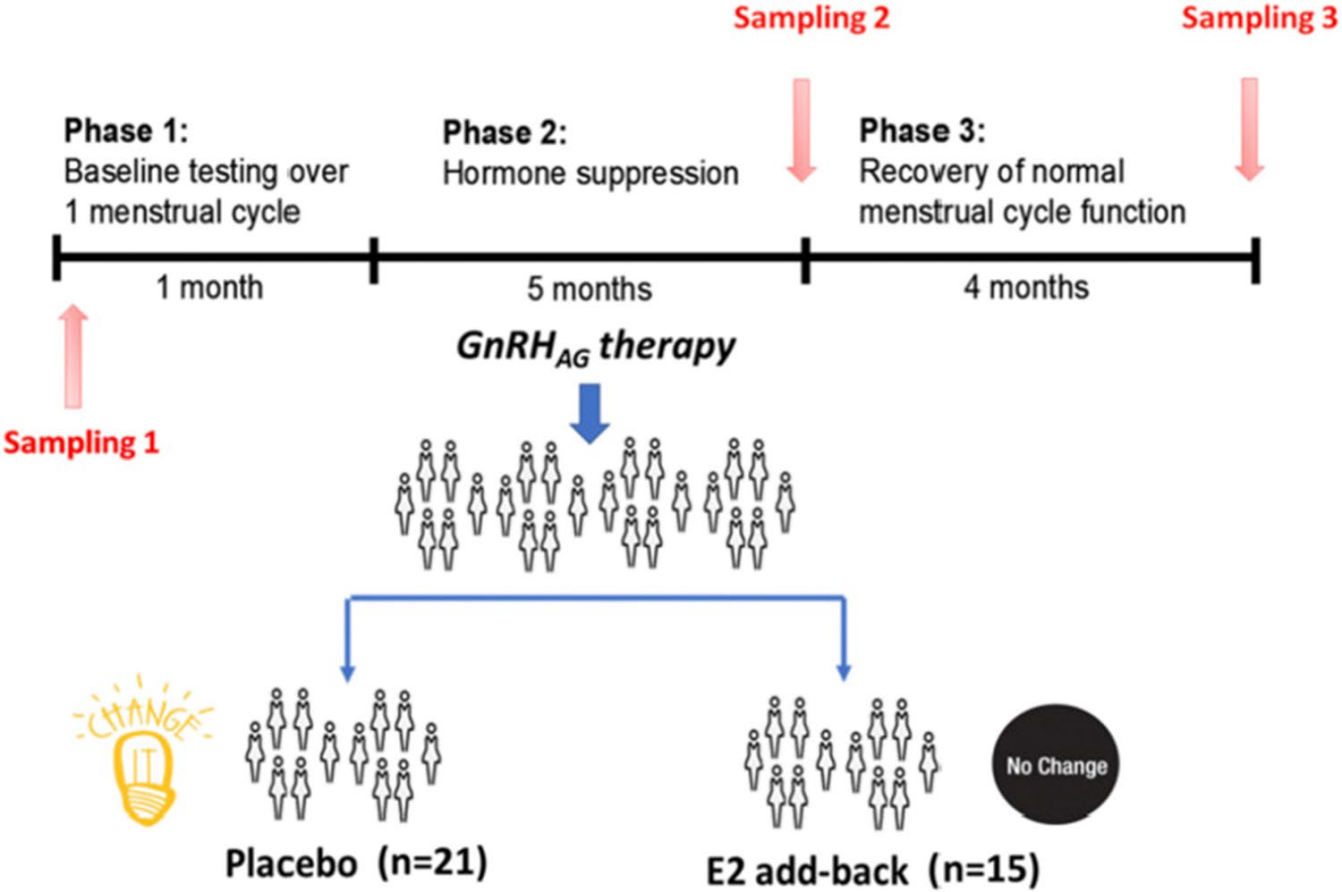
Design of the gonadal hormone suppression intervention study

IgG N-glycans were measured at three time points: pre-intervention, post-intervention and after subsequent recovery (see Fig. 9). GlycanAge expressed in years was calculated using the following formula:

Glycan age = 56.08 + 776.01 × GP6 − 5376.83 (GP6)2 − 215.10 × GP14 − 30.70 × GP15.

where GP < *n* > is *n*th peak in chromatogram expressed as the proportion of total chromatogram area [150]. Suppression of gonadal hormones resulted in a significant increase in glycan age by 9.1 years; however, glycan age was significantly prevented by estradiol therapy (change in glycan age =  − 0.23 years). After the recovery period, glycan age returned to baseline values in both groups.

*Case study three:* Extensive weight loss reduces glycan age by altering IgG N-glycosylation

Bariatric surgery is a considerably effective treatment for severe obesity [151]. To investigate whether weight loss can affect GlycanAge related to inflammation and ageing, an exploratory cohort recruited 37 obese participants from Oxford University Hospitals to the Gastrointestinal Illnesses study, and a replication cohort included 2146 participants from the TwinsUK study. In the exploratory cohort, participants were subjected to 3-week low-calorie diet, followed by bariatric surgery. The IgG N-glycans were chromatographically profiled at baseline, the day of the surgery, and the day of 20% of weight loss. After glycan data quality control, plasma-derived IgG N-glycan traits of 3742 samples with body mass index (BMI) information were longitudinally monitored in the replication cohort [152].

The results showed that the levels of IgG bisecting GlcNAc were significantly decreased (− 0.2801, *P* = 0.0341) after the low-calorie diet intervention, indicating a decreased pro-inflammatory potential of the circulating IgG. Statistical analysis revealed extensive alterations in IgG N-glycome following the bariatric surgery during 1-year follow-up. Four tested derived traits were observed marked changes: a significant increase in digalactosylated (0.0275, *P* = 0.0015) and sialylated (0.0193, *P* = 0.0394) glycans, and a substantial decrease in agalactosylated (− 0.0339, *P* < 0.001) and core fucosylated (− 0.0155, *P* = 0.0394) IgG N-glycans. Loss of BMI over a 20-year period in the TwinsUK cohort validated a weight loss-associated increase in the abundance of digalactosylated (0.2004, *P* < 0.001) glycans and decrease in the abundance of agalactosylated (− 0.1048, *P* = 0.0179) and high mannose (− 0.0519, *P* < 0.001) glycans.

In conclusion, individualised SHS assessments are the reliable and robust platform for successful preventive strategies. Thus, individualised preventive measures for SHS should be based on the established SHS measurements, including SHSQ-25 and IgG N-glycans profiling. The examples of targeted preventive measures, as reviewed above, demonstrated positive effects on the health improvement by applying a combination of subjective and objective SHS measurements. It offered a comprehensive strategy for the individualised preventing from SHS moving into disease phase, from the perspectives of PPPM.

## Treatment algorithm tailored to individual at risk of non-communicable diseases

The innovative PPPM, as the central part of efforts in healthcare management, is aimed at reducing the incidence and prevalence of several non-communicable diseases such as diabetes mellitus, preeclampsia, cardiovascular diseases, mental disorders, malignancies, dental and other pathologies via the SHS concept as one of its pillars.

### Treatment algorithm tailored to individual at risk of T2DM

PPPM holds the key to transforming diabetes mellitus care by promoting adequate patient stratification and prediction of adverse drug-drug interaction [13, 153–155]. Taken together, this led to better health outcomes, delayed the onset of complications, improved quality of life and promoted healthy longevity. As with many chronic diseases, screening for prediabetes is central in PPPM, and it provides the stimulus for initiating treatment and delaying long-term complications and development of diabetes mellitus. SHS is found to be an independent risk factor for type 2 diabetics mellitus (T2DM), which was accompanied by abnormal biochemical levels and poor lifestyle modifications [25, 26]. A Ghanaian cohort study by Adua et al. [25] primarily sedentary (2.97-fold increase), high systolic blood pressure (1.86-fold increase), diastolic blood pressure (2.39-fold increase), and high triglyceride concentration were found to be independent risk factors of SHS. Another cohort by Ge and colleagues amongst a Chinese population the fourth, third, and second quartile SHS scores were found to be associated with a 1.7-fold, 1.6-fold, and 1.5-fold risk of developing T2DM, respectively when compared with the lowest quartile of SHS scores [26]. In the latter study, SHS yielded an area under the curve (AUC) of 0.848 indicating that SHS screening can identify over 80% of prediabetics who may need to be managed. Thus, the management of T2DM is suboptimal; however, undiagnosed risk factors remain prevalent. Identifying SHS individuals at increased risk of poor lifestyle, abnormal clinical characteristics, and cardiometabolic factors creates an opportunity for clinicians to adopt a nutritional and lifestyle modifications tailored towards the high-risk population to prevent the onset of T2DM (2). Early detection followed by appropriate intervention for SHS individuals is important for preventing the onset of T2DM. It is, therefore, incumbent for clinicians to shift from the perspective of delayed intervention approach to early screening of individuals for SHS, especially prediabetes. Routine SHS screening can be useful for the purposes of PPPM.

#### Treatment algorithm tailored to conditions associated with women’s health

Integration of SHS evaluation as a criterion for prediction of preeclampsia has been strongly recommended for healthcare management in pregnancy [9]. In a prospective cohort study conducted amongst a Ghanaian population, SHS identified approximately 89.8% (AUC = 0.898) of normotensive pregnant women within their early gestation to be at risk of developing preeclampsia (PE) and between 78.0 and 82.0% for those likely to develop PE coexisting with stillbirth, intrauterine growth restriction, haemolysis elevated liver enzymes and low platelet count (HELLP) syndrome, acute kidney injury, and dyslipidaemia [31]. Again, these normotensive pregnant women were found to have imbalances in oxidative stress (OS) and angiogenic growth mediators (AGMs) at early gestation [10]. Anto et al. (32) in their study further found that the first quartile for placental growth factor (2.79-fold increase) and vascular endothelial growth factor-A (5.35-fold increase), and the fourth quartile for soluble endoglins (4.31-fold increase), soluble tyrosine kinase receptor 1 (1.84-fold increase), 8-prostagliandinF2-alpha (2.23-fold increase), and 8-hydroxydeoxyguanosine (1.90-fold increase) were independently associated with SHS. While these OS and AGMs are important factors in placental and foetal development, a high SHS score at early gestation in pregnancy is suggestive of increased risk of adverse pregnancy outcome and imbalance in biochemical measures, which calls for combined antioxidant and proangiogenic supplementations tailored towards the high-risk women. Evaluation of SHS criterion along with oxidative stress and angiogenic growth mediators can create an opportunity for predictive preventive and personalised medical care for pregnant women.

Hormonal imbalance, which occurs amongst women during their climacteric period, has been linked to vaginal dryness (VD), even though suboptimal health condition has been implicated [156]. Some of factors that contribute to VD factors including Flammer syndrome (FS) are clearly preventable and, therefore, if managed well, have a potential to reduce the risk of VD. Improving modifiable risk factors associated with FS phenotype at the level of primary prevention and treatment is strongly recommended. SHS assessment can create opportunity for monitoring and management of women likely to develop VD. Thus, future study may screen both pre-and post-menopausal women for VD using SHS criterion.

### Treatment algorithm tailored to individual at risk of poor cardiovascular health

Maintaining a higher ideal cardiovascular health (CVH) is essential for reducing the risk of cardiovascular diseases (CVDs) [23]. In a cross-sectional analysis of a China suboptimal health cohort study (COACS), the prevalence of SHS was 7.10%, 9.18%, 10.04%, and 10.62% in the first, second, third and fourth quartiles of cardiovascular health (CVH) metrics [12]. While the development of CVD is chronic, early identification of SHS individuals with poor CVH metrics such as high blood pressure, high cholesterol, high glucose, cigarette smoking, obesity, physical inactivity and poor dietary habit is an avenue to start personalised medical care [23]. SHS has not only been found to be associated with poor CVH [25, 29] and behavioural factors [29, 88], but also combined assessment of SHS and index of endothelial function and arterial stiffness, independently identified individuals at risk of cardiovascular disease [24]. Thus, in designing the treatment strategies for tailored towards individuals with poor CVH, an algorithm of SHS along with ideal CVH metrics assessment could be an effective preventive strategy for CVD, from the perspective of PPPM.

### Treatment algorithm tailored to individual at risk of mental health

Mental health complaint is one of central domain of SHS. Associations of SHS with psychological symptoms [157] reduced cognitive function [153] and increased incidence of self-reported suicidal ideation [154] has been reported amongst a Chinese population. In a cross-sectional study, prevalence of SHS was 21.0% amongst college students and the independent factors such as somatisation (aOR) = 3.185, 95% CI (2.048–4.953), obsessive–compulsive (aOR = 3.518, 95% CI (2.834–4.368), interpersonal sensitivity (aOR = 1.883, 95% CI (1.439–2.463), and depression (aOR = 1.847, 95% CI (1.335–2.554) were significantly associated with SHS students [157]. In another study, SHS Chinese individuals had approximately threefold increased odds of cognitive impairment. Early signs of high SHS score with poor cognitive, behavioural, and emotional wellbeing calls for the need to initiate preventive and personalised medical care against mental disorders. While the aetiology of poor mental health complaints is multifactorial, ineffective management may lead to irreversible damage and degeneration of neuronal systems [7, 155, 158]. Hence, PPPM is the way for future medical care for mental illness.

### Treatment algorithm tailored to cancer management

Globally, prostate cancer is the one of the multifactorial cancers in men. Whilst the aetiology is multifactorial, improving diagnostics and treatments in the prostate cancer management causes an impressive divergence between, on one hand, permanently increasing numbers of diagnosed prostate cancer cases and, on the other hand, stable or even slightly decreasing mortality rates [4]. However, time of prostate cancer diagnosis is usually delayed. The emergence of PPPM is the way forward for medical approach as this concept can early identify men who may have benign symptoms and are at risk of developing cancers. Prostate cancer patients are benefiting a lot from personalisation of medical services: the general approach by the radical castration has been revised for several subtypes of prostate cancer, since keeping urinary and sexual functions intact allows for significantly higher quality of life for many prostate cancer patients without diminishing the survival rates [4].

Hepatocellular carcinoma (HCC) is ranked as the fifth most common cancer but the second leading cause of all cancer-related mortalities [159]. This has been due to lack of effective screening programs and consequently late diagnosis, multifactorial origin with cumulative risk factors, complex carcinogenesis, tumour heterogeneity, unpredictable impacts of individual microenvironment on tumour development and progression, and, as the consequence, frequently untargeted therapy and cancer resistance towards currently applied treatment approaches [159]. Currently the “wait and treat” approach is inappropriate in the overall hepatocellular carcinoma management. Since SHS has been associated with several non-communicable diseases, a treatment algorithm tailored towards individual at risk of prostate cancer and hepatocellular carcinoma would require prior assessment of SHS. Thus, urgent need in paradigm change towards PPPM is the suggested approach. Since early detection followed by appropriate intervention is important for prevention from the onset of diseases, it is demanded for clinicians to shift from the perspective of delayed intervention approach to early screening of individuals with SHS [31]. Thus, treatment algorithms tailored towards individuals, as suggested in PPPM is the future approach to reducing the risk of non-communicable diseases.

## Outlook: conventional and traditional medicine – a “Hand-in-Hand” collaboration benefiting the patient and healthcare at large

*Modern medicine*, also referred to as “Western medicine”, “standard medicine”, or “conventional medicine” is “evidence based” to penetrate phenotypes and to unravel the transcendent truth, and it has been nourished by the constant tension between the unknown and known, and imperfect and perfect [160, 161]. Modern medicine has been committed to the establishment of the standardised guidelines for the diagnosis followed by the prescription of therapies to alleviate symptoms of disease [162]. However, the same diagnosis can derive from various molecular pathologies; therefore, different therapeutic strategies may be optimal for each individual patient. Furthermore, if the alteration in molecular pathways for the disease can be identified at an early stage, then corrective measures can be initiated even before any symptoms of the disease develop, resulting in either delayed onset, or complete prevention of the disease from the perspectives of PPPM [18].

*Traditional *medicine, also called “Eastern medicine”, “complementary medicine” or “alternative medicine”, is “experience based” to pay more attention to the overall state of the individual, including general stress and physical and psychological conditions [18]. The WHO defines traditional medicine as “the sum total of the knowledge, skills, and practices based on the theories, beliefs, and experiences indigenous to different cultures, whether explicable or not, used in the maintenance of health as well as in the prevention, diagnosis, improvement or treatment of physical and mental illness” [163]. Due to the different origins and cultures, traditional medicine comprises a range of ancient, long-standing but still evolving treatment approaches being practised mainly in their countries of origin as well as in countries into which corresponding expertise has been “imported” [164]. In traditional medicine, treatment approaches consist of five categories listed in Table 5 with examples [165].

**Table 5:** Categories with examples of traditional medicine treatment approaches

Category	Example	Definition
Mind–body therapies	Meditation	Focused breathing or repetition of words or phrases to quiet the mind
	Biofeedback	Using simple machines, the patient learns how to affect certain body functions that are normally out of one's awareness (such as heart rate)
	Hypnosis	A state of relaxed and focused attention in which a person concentrates on a certain feeling, idea, or suggestion to aid in healing
	Yoga	Systems of stretches and poses, with special attention given to breathing
	Tai Chi	Involves slow, gentle movements with a focus on the breath and concentration
	Imagery	Imagining scenes, pictures, or experiences to help the body heal
	Creative outlets	Interests such as art, music, or dance
Biologically based practices	Vitamins and dietary supplements	Nutrients that are added to the diet
	Botanicals	plants or parts of plants
Manipulative and body-based practices	Massage	The soft tissues of the body are kneaded, rubbed, tapped, and stroked
	Chiropractic therapy	A type of manipulation of the spine, joints, and skeletal system
	Reflexology	Using pressure points in the hands or feet to affect other parts of the body
Biofield therapy	Reiki	Balancing energy either from a distance or by placing hands on or near the patient
	Therapeutic touch	Moving hands over energy fields of the body
Whole medical systems	Ayurvedic medicine	A system from India in which the goal is to cleanse the body and restore balance to the body, mind, and spirit
	Traditional Chinese medicine	Based on the belief that health is a balance in the body of two forces called “Yin” and “Yang”
	Homoeopathy	Uses very small doses of substances to trigger the body to heal itself
	Naturopathic medicine	Uses various methods that help the body naturally heal itself. An example would be herbal treatments

The fundamental principles of most traditional medicine, such as Ayurveda [166], Bush medicine [167], traditional Chinese medicine (TCM) [160], and *Unani Tibb* (“Greek Medicine” in Arabic) [168], are all to seek to invoke and restore balance of the elements of the body. For example, TCM believes that balances of “*Yin*” and “*Yang*” can keep body’s health [33]. The pattern describes the patient’s body as being in a situation of “distress” or “imbalance”, and illnesses arise through the pattern imbalance [33, 161]. Thereby, TCM specifies a terminology of “pattern of disharmony” that gathers and weaves all information about physical and psychological conditions, including the presented symptoms, lost function and adaptability, as well as the patient's other general characteristics [18, 161]. The therapy then attempts to bring the configuration into balance, to restore harmony to the individuals’ [160]. However, traditional medicine lacks the molecular mechanisms and scientific proofs for the therapeutic effects on patients.

### Integrative medicine — Eastern medicine meets Western medicine

**Fig. 10:**
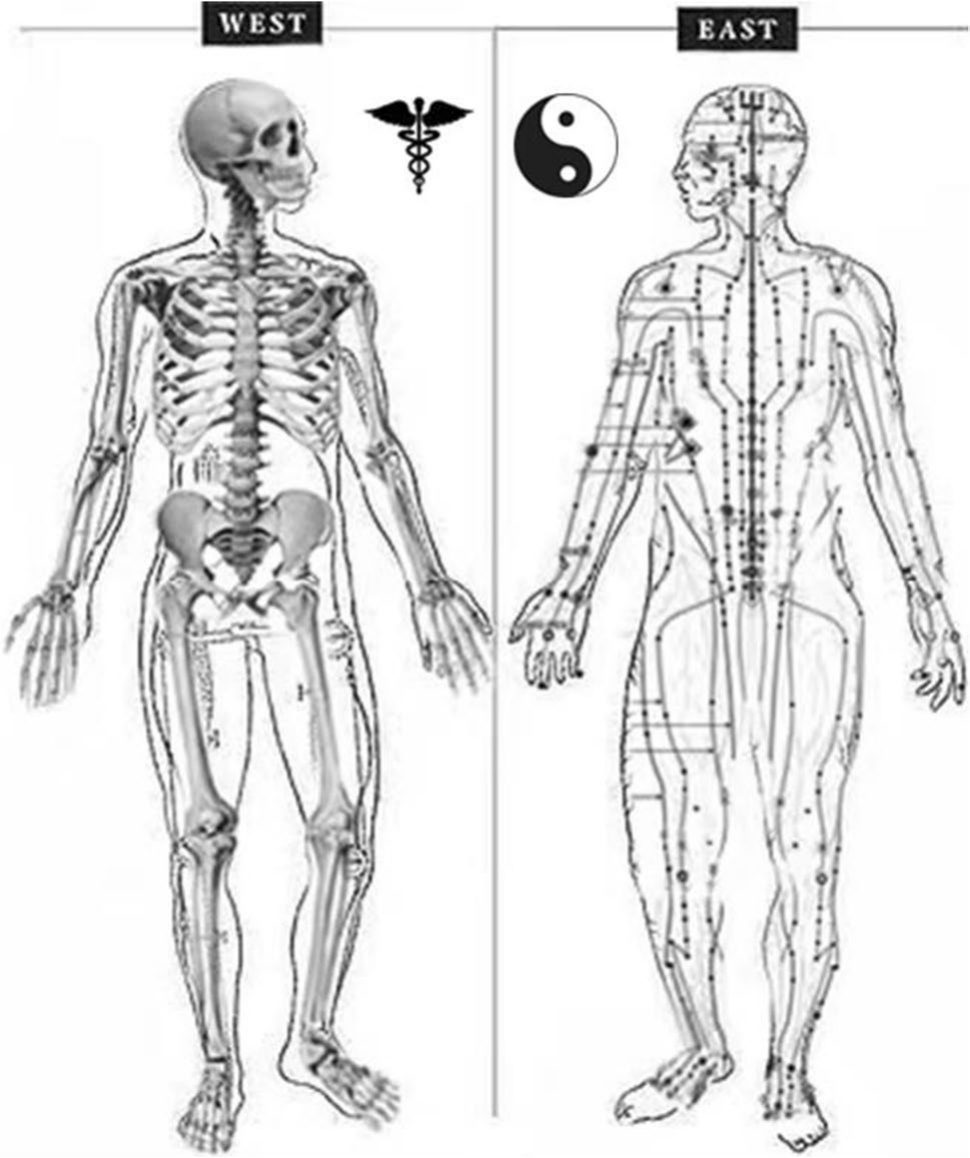
Western medicine meets Eastern medicine — acting hand-in-hand in the frame-work of Predictive, Preventive and Personalised Medicine (3PM/PPPM); skeleton (left). Meridian and acupoints (right)

*Integrative *medicine is an approach of combining modern and traditional medicine in a coordinated way that has been scientifically proven to be safe and effective [165, 169]. This approach emphasises the patient’s preferences while also it attempting to address the mental, physical, and spiritual elements of wellness and health [165]. Integrative health additionally stresses multimodal interventions, which are at least two interventions like conventional medicine, health-promoting lifestyle, physical rehabilitation, psychotherapy, and complementary health techniques in various combinations, with an emphasis on treating the whole person rather than, for example, one organ system. Integrative health strives to achieve well-coordinated care across various providers and institutions by combing conventional and complementary approaches to really focus in the general individual [170] (Fig. 10).

With the goal of building the bridge between modern and traditional medicine, we coined the concept of “Suboptimal Health Status” (SHS) via the unique expertise of TCM to address the intermediate physical condition between health and disease from the viewpoint of PPPM [18, 20]. Meanwhile, by combining the merits of both modern medicine and TCM principles, we developed a reliable, valid and robust tool called the “SHSQ-25” to measure SHS and support greater personalisation in preventative health care [21, 27, 29]. Further we achieved a linguistically valid translation of the SHSQ-25 into Arabic, Chinese, English, Japanese, Korean, Portuguese, Russian, and Thai to overcome language barriers and enhance the power of the tool for further expanded application in a greater number of populations. The innovative hybrid approaches utilise advantages of both modern medicine and TCM; furthermore, the combination of these two disciplines benefit patients and enrich the spectrum of tools and overall expertise of the dedicated professional groups assuring the reproducibility of TCM technologies and outcomes [15].

SHS is a new health dimension for translational medicine via the practical application of the SHSQ-25 in the general population [20]. Studies have demonstrated that SHS is associated with an alteration of intestinal microbiota [171], telomere length [63], mRNA expression level of glucocorticoid receptor α (GRα) [67], cardiovascular health metrics [23], plasma cortisol [172], plasma catechol-amines [67], oxidative stress [32], blood transcriptome profiling [173], metabolites [118], plasma N-glycans profiling [70], and biological ageing [63], with an increased incidence of cardiovascular diseases, type 2 diabetes mellitus (T2DM) [26], metabolic syndrome (MetS) [70], cancer [120], Parkinson’s disease [121, 122], systemic lupus erythematosus (SLE) [123], rheumatoid arthritis (RA) [124], dyslipidemia [125], hypertension [126], stroke [126], and preeclampsia [31]. Thereby, SHS plays an important role in the prediction of NCDs pathogenesis and progression.

Previous studies have revealed that SHS is mainly affected by lifestyle factors [174]. With the rapid advancement of technology, changes in the environment, and people’s lifestyles, the risk of SHS has steadily increased. Meanwhile, people are dealing with personal, familial, and societal stress, as well as a variety of obstacles including changes in their living environment, overwork, complex interpersonal relationships, sleep deprivation, excessive psychological stress, unbalanced diet, and inadequate exercise [175]. Thus, PPPM strategies and prognosis for SHS are expected to become increasingly important. In the field of TCM, the mainstream and accepted forms of therapy include acupuncture, dietary therapy, herbal medicine, moxibustion, massage, qigong (e.g., *Tai Chi, Baduanjin*, and *Wuqinxi*) applied singularly or in combination to treat and prevent diseases or maintain wellbeing [176, 177]. For instance, the evidence-based TCM treatment for SHS proved that *Baduanjin* exercise (one of the most common forms of *Qigong* consisting of eight separate, delicate, and smooth exercise movements) [135], moxibustion (a form of heat therapy in which dried plant materials called “moxa” are burned on acupoints) [138], and cupping (application of heated cups on the skin to remove stagnation and stimulate the flow of “*Qi*”) [142] are valuable approaches for preventing SHS.

In conclusion, the early identification and prevention of SHS may both minimise prospective risk factors for SHS. This has the potential to not only improve the measures of the individuals’ quality of life and health outcome, but also serve to decrease the economic burden that non-communicable diseases place on healthcare systems worldwide. This successful hand-in-hand, modern meets tradition, west meets east, pattern of health care provides a comprehensive and holistic approach that benefits both individuals and global health care systems, as realised through the concepts of PPPM.

## Concluding remarks and expert recommendations

The absolute majority of expanding non-communicable disorders do carry a chronic character, over a couple of years progressing from reversible suboptimal health conditions to irreversible severe pathologies and cascading collateral complications. The time-frame between onset of SHS and clinical manifestation of associated disorders is the operational area for an application of reliable risk assessment tools and predictive diagnostics followed by the cost-effective targeted prevention and treatments tailored to the person. Particularly in adolescence, the adverse health effects by suboptimal health carry reversible character. However, this unique capacity to repair health damage and to establish sustainable health supportive lifestyle habits is not adequately propagated and not utilised by currently applied concepts of reactive medical services [5].

This article demonstrates advanced strategies in bio/medical sciences and healthcare focused on suboptimal health conditions in the frame-work of PPPM. Potential benefits in healthcare systems and for society at large include but are not restricted to an improved life-quality of populations and socio-economical groups, advanced professionalism of healthcare-givers, and sustainable healthcare economy.

Contextually, following programmes are recommended: innovative population screening programmes focused on stratified groups such as adolescents and young adults, planned pregnancies and post-partum, elite athletes, intentional weight loss, low socio-economic status, and healthy elderly, amongst others.

For advanced risk assessment, purpose-adapted highly effective muti-level diagnostic tools are strongly recommended such as specialised surveys, liquid biopsy and microbiome analysis, multi-omics, and comprehensive individualised patient profiling (CIPP) by application of artificial intelligence (AI, big data management, machine learning) [178–180].

Based on the CIPP, cost-effective preventive measures and treatments tailored to the person can be created considering individual risks and optimal medication options including natural substances and their derivatives [181].

The following healthcare areas are proposed to strongly benefit from the above proposed measures:

- Stress overload associated pathologies [156, 158, 182–184]
- Planned pregnancies [8]
- Periodontal health [185]
- Eye disorders [5]
- Inflammatory disorders, wound healing and pain management with associated complications [186, 187]
- Metabolic disorders [2]
- Cardiovascular pathologies [178]
- Cancers [4]
- Stroke, particularly of unknown aetiology and in young individuals [7]
- Sleep medicine [188, 189]
- Individually suboptimal body weight [185, 188]
- Sports medicine [190]
- Male and female health [191]
- Improved individual outcomes under pandemic conditions such as COVID-19 [185, 188, 192–194] amongst others [30, 190, 191].

## Data Availability

Not applicable.

## Abbreviations

ADCC: Antibody-dependent cellular cytotoxicity
AI: Artificial intelligence
AGMs: Angiogenic growth mediators
BMI: Body mass index
CFIDS: Chronic fatigue immune dysfunction syndrome
CFS: Chronic fatigue syndrome
CMI: Cornell Medical Index
COPD: Chronic obstructive pulmonary diseases
COR: Serum cortisol
CVD: Cardiovascular diseases
DBP: Diastolic blood pressure
EPMA: European Association for Predictive, Preventive and Personalised Medicine
EXC: Exercise intervention
FcyRIIB: Fc gamma receptor type IIB
FS: Flammer syndrome
GHQ-12: 12-Item General Health Questionnaire
GlcNAc: N-acetylglucosamine
GLU: Plasma glucose
GR: Glucocorticoid receptor
HCC: Hepatocellular carcinoma
HDLC: High-density lipoprotein cholesterol
HF: High-frequency
LDLC: Low-density lipoprotein cholesterol
LF: Low-frequency
LMICs: Low- and middle-income countries
MBL: Mannose binding lectin
ME: Myalgic encephalomyelitis
NCDs: Non-communicable diseases
OS: Oxidative stress
PE: Preeclampsia
PVFS: Post-viral fatigue syndrome
REC: Recovery period
RTL: Relative telomere length
SBP: Systolic blood pressure
SHS: Suboptimal health status
SHSC: Suboptimal Health Study Consortium
SHSQ-25: Suboptimal health status questionnaire-25
TC: Traditional cupping
TCH: Total cholesterol
TCM: Traditional Chinese medicine
TG: Triglyceride
T2DM: Type 2 diabetes mellitus
UMS: Unexplained medical syndrome
VD: Vaginal dryness
WHOQOL-100: World Health Organisation Quality of Life-100
WL: Wait-list
3PM/PPPM: Predictive, Preventive and Personalised Medicine
